# Engineering applicability of coarse *Prunus spinosa* biochar for post‑nitrification ammonium polishing in recirculating aquaculture systems

**DOI:** 10.1038/s41598-026-44237-6

**Published:** 2026-04-27

**Authors:** Ali Asghar Najafpoor, Hadi Farsiani, Mojtaba Davoudi, Fatemeh Kariminejad

**Affiliations:** 1 Department of Environmental Health Engineering, School of Health, Mashhad University of Medical Sciences, Mashhad, Iran; 2 Social Determinants of Health Research Center, Mashhad University of Medical Sciences, Mashhad, Iran; 3 Antimicrobial Resistance Research Center, Basic Sciences Research Institute, Mashhad University of Medical Sciences, Mashhad, Iran; 4 Student Research Committee, Department of Environmental Health Engineering, School of Health, Mashhad University of Medical Sciences, Mashhad, Iran

**Keywords:** Recirculating aquaculture systems, Post‑nitrification ammonium polishing, Prunus spinosa biochar, Fixed‑bed adsorption, Nitrogen management, Biotechnology, Environmental sciences

## Abstract

Residual ammonium following biological nitrification remains a critical operational concern in recirculating aquaculture systems (RAS), where even low concentrations can induce chronic toxicity and destabilize system performance. Under post-nitrification conditions, engineering reliability, hydraulic compatibility, and material reusability often outweigh maximum adsorption capacity. This study evaluates the applicability of coarse-grained *Prunus spinosa* biochar (2–4 mm) as a supplementary polishing medium for residual ammonium control downstream of biological nitrification. Batch adsorption experiments were conducted under RAS-relevant conditions (pH 6.8–7.2; 15–35 °C) to quantify ammonium uptake behavior, selectivity in the presence of competing nitrogen species, and governing kinetic, equilibrium, and thermodynamic mechanisms. The biochar exhibited selective ammonium removal, achieving up to 5% uptake, while nitrite and nitrate removal remained below 1%, preserving nitrification functionality. Ammonium adsorption followed pseudo-second-order kinetics and was well described by the Langmuir model (R^2^ > 0.98), with moderate monolayer capacities (3.24–3.57 mg g^−1^). Thermodynamic analysis confirmed spontaneous and endothermic adsorption, with increased favorability at elevated aquaculture-relevant temperatures. To address engineering feasibility beyond batch conditions, short-term continuous-flow fixed-bed column experiments were performed, demonstrating stable hydraulic operation and preferential ammonium removal. Biochar reusability was confirmed over five adsorption–regeneration cycles using 0.1 M NaCl, with 9.1% efficiency retention. Overall, the results demonstrate that coarse *Prunus spinosa* biochar is a technically viable, reusable, and cost-effective ammonium polishing medium for RAS, providing an application-oriented foundation for future long-term and pilot-scale fixed-bed studies.

## Introduction

 Aquaculture is among the fastest‑growing food production sectors worldwide, playing a critical role in global food security and nutrition^1^. However, intensive aquaculture practices generate nutrient‑rich effluents containing nitrogenous compounds, particularly ammonium (NH_4_^+^), nitrite (NO_2_^−^), and nitrate (NO_3_^−^), which pose significant risks to aquatic ecosystems and system performance^2^. Elevated ammonium concentrations are especially problematic due to their direct toxicity to fish and invertebrates, impairment of gill function, and destabilization of biological filtration units in recirculating aquaculture systems (RAS)^3–5^. Consequently, maintaining total ammonia nitrogen (TAN) below regulatory and species‑specific safety thresholds is essential for both environmental protection and sustainable aquaculture operation^6,7^.

In modern RAS, biological nitrification represents the primary process for oxidizing ammonium to nitrate, thereby enabling high water recirculation rates with reduced freshwater demand^6,8^. Nevertheless, nitrification alone often fails to ensure consistently low residual ammonium (NH_4_^+^) concentrations under fluctuating feeding loads, seasonal temperature variations, or transitional operational states such as start‑up or biomass aging^9–12^. Even low residual ammonium concentrations (< 5 mg L^−1^) can lead to unsafe levels of unionized ammonia (NH_3_), particularly at elevated pH and temperature, posing chronic toxicity risks for sensitive species^13^. Accordingly, supplementary downstream treatment steps are frequently required to stabilize effluent quality and enhance system resilience. In this context, fixed‑bed adsorption units represent an attractive configuration for post‑nitrification polishing due to their continuous operation, low energy demand, and ease of integration into existing RAS infrastructure^14–16^.

Conventional tertiary treatment technologies—such as ion exchange resins, membrane filtration, advanced oxidation, and chemical polishing—can effectively reduce residual nitrogen but often involve high capital costs, operational complexity, or intensive maintenance requirements, limiting their practicality for small‑ to medium‑scale aquaculture facilities^17,18^. Adsorption‑based processes offer a simpler and more flexible alternative, particularly when implemented using low‑cost, biomass‑derived sorbents capable of operating under fluctuating water quality conditions^19,20^.

Among such materials, biochar has attracted increasing interest due to its porous structure, surface functional groups, ion‑exchange capacity, and compatibility with circular‑economy principles^21,22^. However, a critical research gap persists in the translation of laboratory-scale biochar adsorption studies to practical engineering applications. While numerous investigations have demonstrated the ammonium adsorption capacity of various biochars, the majority have employed finely powdered materials optimized for maximum equilibrium uptake under idealized batch conditions^23–27^. This approach, although valuable for fundamental mechanistic understanding, overlooks the practical constraints associated with real-world implementation in recirculating aquaculture systems. Specifically, the use of fine biochar powders presents substantial operational challenges including hydraulic instability, excessive pressure drops, particle escape, and difficulties in recovery and regeneration—issues that are particularly problematic in continuous-flow fixed-bed configurations essential for RAS polishing units^28,29^. Consequently, there exists a significant disconnect between the extensive literature on biochar adsorption capacity and the engineering requirements for viable fixed-bed polishing applications^30^. In contrast, coarse‑grained biochar materials inherently sacrifice some adsorption capacity but offer substantial advantages in terms of mechanical robustness, ease of separation, regeneration potential, and compatibility with fixed‑bed configurations—attributes essential for real‑world RAS implementation^31^. These trade‑offs highlight a persistent gap between laboratory‑scale adsorption studies and their translation into practicable fixed‑bed polishing units.

Accordingly, the present study focuses on post‑nitrification ammonium polishing under low residual NH_4_^+^, NO_2_^−^, and NO_3_^−^ levels representative of mature recirculating aquaculture system (RAS) effluents, rather than primary nitrogen removal. To explicitly address the persistent, disconnect between mechanistic adsorption studies and engineering feasibility, biochar derived from Prunus spinosa (PS)—a locally available lignocellulosic feedstock with favorable mineral composition—was investigated within a deliberately application‑oriented framework^32,33^. In contrast to the prevailing emphasis on finely powdered or chemically modified biochars optimized for maximum equilibrium capacity^30,34^, a coarse particle fraction was intentionally selected to prioritize hydraulic compatibility, mechanical robustness, regeneration potential, and operational practicality for prospective fixed‑bed integration, accepting reduced surface area as an informed and necessary design trade‑off.

Although long‑term fixed‑bed performance and full breakthrough behavior were not the primary focus of this study, the experimental framework is intended to characterize material suitability and engineering applicability rather than to demonstrate an operational fixed‑bed system. Laboratory‑scale batch experiments were intentionally employed as a foundational step to generate mechanistic, thermodynamic, and design‑relevant parameters that are routinely required prior to fixed‑bed process translation^35,36^. The specific objectives of this study were therefore to: (1) quantify the adsorption performance and selectivity of coarse‑grained PS biochar toward NH_4_^+^ in the presence of competing NO_2_^−^ and NO_3_^−^ under conditions representative of post‑nitrification RAS effluents; (2) elucidate governing adsorption mechanisms through kinetic, equilibrium, and thermodynamic analyses across RAS‑relevant pH and temperature ranges; and (3) evaluate the engineering applicability of the material for post‑nitrification polishing by examining process stability, structural integrity, and preliminary fixed‑bed performance relative to internationally accepted water‑quality thresholds.

To the authors’ knowledge, this study represents one of the first systematic evaluations to explicitly position coarse‑grained biochar as a post‑nitrification ammonium polishing medium under low residual NH_4_^+^, NO_2_^−^, and NO_3_^−^ conditions, where engineering feasibility, operational stability, and fixed‑bed compatibility are prioritized over maximum adsorption capacity.

## Materials and methods

### Study framework and experimental design

This experimental study investigated the adsorption behavior of *PS* biochar for the removal of inorganic nitrogen species—ammonium (NH_4_^+^), nitrite (NO_2_^−^), and nitrate (NO_3_^−^)—from aquaculture‑relevant aqueous matrices. All experiments were conducted under controlled laboratory conditions using batch systems. Batch operation was intentionally selected to isolate intrinsic adsorption behavior, eliminate hydrodynamic interference, and generate fundamental kinetic, equilibrium, and thermodynamic parameters required for evaluating feasibility and predicting material performance in fixed‑bed polishing applications^30^.

To reflect realistic operational conditions encountered in RAS, adsorption performance was evaluated at three temperatures: 15 °C, 25 °C, and 35 °C. These temperatures correspond to typical seasonal ranges experienced in cold‑water and warm‑water aquaculture facilities and allow assessment of thermal sensitivity and process stability under practically relevant conditions^37^. While the primary experimental framework relied on batch systems to isolate intrinsic adsorption behavior, complementary fixed‑bed column experiments were subsequently conducted to preliminarily examine hydraulic compatibility and early‑stage performance under continuous flow conditions.

### Biochar sourcing, preparation, and physicochemical characterization

Biochar derived from *PS* wood was commercially obtained from Fasl‑e‑Panjom Science and Technology Park (Shiraz, Iran). The biochar was produced via slow pyrolysis at 500 °C under an inert nitrogen atmosphere, yielding a carbon‑rich material with a stable porous structure.

Prior to use, the biochar was sieved to obtain particles in the 2–4 mm size range, washed thoroughly with deionized water to remove loose ash and impurities, and oven‑dried at 105 °C for 24 h. This coarse particle fraction was deliberately selected to simplify recovery, minimize secondary particle release, maintain mechanical integrity during handling and regeneration, and ensure hydraulic compatibility with potential fixed‑bed operation. Although finer fractions typically provide higher adsorption capacities, this design choice intentionally prioritizes operational feasibility and long‑term stability over surface‑area maximization^31^.

The surface chemistry of the biochar was characterized using Fourier-transform infrared spectroscopy (FTIR) (Thermo Avatar; 400–4000 cm^−1^) via the KBr pellet method to identify functional groups involved in nitrogen adsorption. Structural ordering and crystalline phases were examined by X-ray diffraction (XRD) (Philips PW1730) employing Cu Kα radiation (λ = 1.5406 Å) across a 2θ range of 10–80°.

Electrostatic surface properties were evaluated through zeta potential measurements conducted in 10 mM KCl solution at pH 7.0 using a Horiba Zetasizer. Textural characteristics, including specific surface area, pore volume, and pore size distribution, were determined using the Brunauer–Emmett–Teller (BET) method based on N_2_ adsorption–desorption isotherms at 77 K (BELSORP Mini II). Elemental composition (C, H, N, and S) was quantified by CHNS analysis (ECS 4010), and complementary inorganic elemental analysis was carried out using inductively coupled plasma optical emission spectroscopy (ICP-OES) (VISTA-PRO).

All physicochemical analyses were performed by Mahamax Company (Tehran, Iran), a certified provider of laboratory and analytical services.

### Batch adsorption experiments

Batch adsorption experiments were designed to generate equilibrium and kinetic parameters required for process evaluation and material screening, rather than to simulate continuous fixed‑bed operation. Synthetic aqueous solutions of NH_4_^+^, NO_2_^−^, and NO_3_^−^ were prepared individually using analytical‑grade NH₄Cl, NaNO_2_, and KNO_3_, respectively. Stock solutions (1000 mg L^−1^) were diluted to initial concentrations ranging from 10 to 200 mg L^−1^, encompassing both stressed conditions and low residual concentrations typical of post‑nitrification RAS effluents^38^. In particular, batch tests conducted at 10–50 mg L^−1^ were considered representative of residual or transitional NH_4_^+^, NO_2_^−^, and NO_3_^−^ levels that may occur in otherwise mature RAS during short‑term operational fluctuations, whereas lower concentrations were used to directly inform polishing‑level performance.

All experiments were conducted in 250 mL Erlenmeyer flasks containing 100 mL of solution, with biochar dosages ranging from 2 to 20 g L^−1^. All nitrogen species concentrations are reported on an ionic basis (mg L^−1^).

The pH was maintained within a near‑neutral range (6.8–7.2) using dilute NaOH or HCl to minimize pH‑induced speciation effects and ensure compatibility with aquaculture reuse scenarios^39^. Flasks were agitated at 150 rpm for 24 h, a duration sufficient to achieve adsorption equilibrium. Following equilibration, suspensions were filtered, and equilibrium concentrations were analyzed using standard spectrophotometric methods. Adsorption performance was expressed in terms of removal efficiency (R, %) and equilibrium adsorption capacity (q_e_, mg g^−1^), calculated as follows^40^:

1 $$R\left(\%\right)=\frac{{C}_{0}-{C}_{e}}{{C}_{0}}\times100$$

2 $${q}_{e}=\frac{({C}_{0}-{C}_{e})V}{m}$$

where C_0_ and Ce (mg L^−1^) denote the initial and equilibrium solute concentrations, V (L) is the solution volume, and m (g) is the mass of biochar.

### Kinetic modeling

Kinetic analyses were performed to elucidate adsorption rates and to identify the dominant mass-transfer and surface interaction mechanisms governing nitrogen uptake by the biochar. The application of multiple kinetic models enables differentiation between diffusion-controlled processes and surface-reaction-controlled adsorption, as well as between physical and chemical binding mechanisms^41^.

The pseudo-first-order model was applied under the assumption that adsorption is primarily governed by physisorption:

3 $$\mathrm{ln}\left({q}_{e}-{q}_{t}\right)=\mathrm{ln}{q}_{e}-{k}_{1}t$$

where k_1_ (min^−1^) is the adsorption rate constant.

The pseudo-second-order model, often associated with chemisorption processes involving electron sharing or exchange, was described by:

4 $$\frac{t}{{q}_{t}}=\frac{1}{{k}_{2}{q}_{e}^{2}}+\frac{t}{{q}_{e}}$$

where k_2_ (g mg^−1^ min^−1^) is the pseudo‑second‑order rate constant.

To further assess diffusion limitations, the Weber–Morris intraparticle diffusion model was applied:

5 $${q}_{t}={k}_{p}{t}^{0.5}+C$$

where kp (mg g^−1^h^−0.5^) indicates the intraparticle diffusion rate and C (mg g^−1^) reflects the contribution of boundary-layer resistance^42^.

### Isotherm modeling

Equilibrium adsorption behavior was evaluated using Langmuir, Freundlich, and Temkin isotherm models to characterize the interaction between nitrogen species and the biochar surface under equilibrium conditions.

The Langmuir isotherm, which assumes monolayer adsorption on a homogeneous surface with finite binding sites, is expressed as:

6 $${q}_{e}=\frac{{q}_{m}{K}_{L}{C}_{e}}{1+{K}_{L}{C}_{e}}$$

Adsorption favorability was assessed using the dimensionless separation factor:

7 $$\mathrm{RL} = 1/(1 + \mathrm{K}_{\mathrm{L}}\mathrm{C}_{0})$$

The Freundlich isotherm, representing heterogeneous surface adsorption, was defined as:

8 $${q}_{e}={k}_{F}{tC}^{1/n}$$

where 1/n values between 0 and 1 indicate favorable adsorption.

The Temkin isotherm accounts for adsorbent–adsorbate interactions and a linear decrease in adsorption energy with surface coverage:

9 $${q}_{e}=B\mathrm{ln}ln{A}_{T}+Bln{C}_{e}$$

10 $$B=\frac{RT}{{b}_{t}}$$

The combined application of these models provides complementary insight into adsorption mechanisms not discernible from removal efficiencies alone^43^.

These models were selected to provide parameters commonly used for preliminary fixed-bed design and performance prediction under low-strength influent conditions.

### Thermodynamic analysis

Thermodynamic parameters were determined to evaluate the energetic feasibility and temperature dependence of nitrogen adsorption onto *PS* biochar. Equilibrium constants derived from Langmuir parameters at 15, 25, and 35 °C were used to calculate standard Gibbs free energy (ΔG°), enthalpy (ΔH°), and entropy (ΔS°) changes according to:

11 $$\mathrm{ln}{k}_{e}=-\frac{\varDelta{H}^{0}}{RT}+\frac{\varDelta{S}^{0}}{R}$$

12 $$\varDelta{G}^{0}=-RTln{K}_{e}$$

where K_e_ = q_e_/Ce. These thermodynamic indicators clarify whether adsorption is spontaneous, endothermic or exothermic, and thermally stable under variable conditions encountered in aquaculture operation^44^.

### Fixed-bed column configuration and operation

A laboratory‑scale up‑flow fixed‑bed reactor was employed to preliminarily assess hydraulic compatibility and early‑stage material behavior under continuous‑flow conditions, rather than to establish full breakthrough performance or long‑term operational metrics^45^. The column was constructed from a cylindrical acrylic tube with an internal diameter of 6 cm and a total height of 40 cm. A fixed biochar bed with a height of 20 cm was established at the center of the column, corresponding to an effective packed‑bed volume of approximately 375 mL. The effective hydraulic volume of the column, including the freeboard region, was approximately 750 mL.

The biochar particle size fraction, identical to that used in batch experiments, was deliberately selected to ensure mechanical stability, minimize pressure losses, and reduce the risk of clogging or particle washout under continuous operation^46^. The column was operated in up‑flow mode to promote uniform hydraulic distribution and to avoid bed compaction during long‑term operation^47^.

Synthetic influent containing ammonium at an initial concentration of 50 mg L^−1^ was continuously supplied to the column using a peristaltic pump at a constant flow rate of 1 mL min^−1^. Under these conditions, the empty bed contact time (EBCT), calculated based on the packed‑bed volume, was approximately 6.25 h, while the hydraulic retention time (HRT), calculated based on the effective hydraulic volume, was approximately 12.5 h^48^.

To eliminate start‑up artifacts and ensure representative effluent measurements, sample collection was initiated only after the elution of the first effluent drop, corresponding to one complete HRT (12.5 h). Effluent samples were subsequently collected at defined time intervals and analyzed for NH_4_^+^, NO_2_^−^, and NO_3_^−^ concentrations using the same analytical methods and quality control procedures applied in batch experiments. The column was operated continuously for up to 144 h. Effluent concentration profiles were recorded over the operational period, and data beyond 24–48 h were not further reported due to the absence of systematic temporal variation.

The fixed‑bed experiment was designed as a complementary engineering evaluation rather than a full breakthrough study. Accordingly, the results are intended to support assessment of material suitability, selectivity, and hydraulic compatibility under low‑strength post‑nitrification conditions, rather than to establish design‑scale breakthrough parameters.

### Regeneration and reuse protocol

Following adsorption experiments, the spent PS biochar was subjected to a regeneration procedure to evaluate its reusability under repeated operational cycles. Regeneration was carried out using an aqueous sodium chloride (NaCl) solution with a concentration of 0.1 M, selected to promote ammonium desorption primarily through ion-exchange mechanisms while avoiding aggressive chemical treatment.

The exhausted biochar was contacted with the regeneration solution under controlled laboratory conditions for a defined period, after which the material was thoroughly rinsed with deionized water to remove residual electrolyte. The regenerated biochar was then dried at ambient conditions and reused in subsequent adsorption cycles following the same experimental procedures applied during the initial adsorption stage.

This regeneration–reuse sequence was repeated over multiple consecutive cycles to assess the cyclic stability of the biochar under repeated adsorption and desorption conditions. All regenerated samples were handled and analyzed using identical protocols to ensure consistency and comparability across cycles.

### Analytical methods

Water quality parameters were quantified following Standard Methods for the Examination of Water and Wastewater^49^. The analytical techniques, instruments, and detection limits employed in this study are summarized in Table 1.

**Table 1:** Analytical methods and limits of detection (LOD) for nitrogen species *.

Parameter	Method description	Standard method number	Instrument model	Detection limit (mg L^−1^)
Ammonia (NH_4_^+^)	Nessler’s colorimetric method (425 nm)	4500-NH_3_	Hach SQ2800 UV–Vis	0.1
Nitrite (NO_2_^−^)	Sulfanilamide–NED colorimetric method (543 nm)	4500-NO_2_^−^	Hach SQ2800 UV–Vis	0.1
Nitrate (NO_3_^−^)	UV spectrophotometric method (220/275 nm)	4500-NO_3_^−^ B	Hach SQ2800 UV–Vis	0.1
pH	Glass electrode	4500-H^+^	Hach HQ440D	-

All nitrogen species are reported on an ionic basis (NH_4_^+^, NO_2_^−^, NO_3_^−^) in units of mg L^−1^. Samples were filtered through 0.45 μm membrane filters prior to analysis. LOD values were determined experimentally in the laboratory.

### Statistical analysis

All batch adsorption experiments were conducted in duplicate, and reported values represent mean results. Error bars are not shown in figures where variability was smaller than symbol size. Replication was performed to verify experimental reproducibility and internal consistency under controlled laboratory conditions. All experimental data were initially processed using Microsoft Excel 2016. Nonlinear regression analyses, kinetic and isotherm model fitting, and graphical visualizations were performed using OriginPro 2024. Model accuracy and goodness of fit were assessed using the coefficient of determination (R^2^), chi-square (χ^2^), and root mean square error (RMSE).

## Results

### Physicochemical characteristics of *PS* biochar

The physicochemical properties of *PS* biochar produced were characterized to describe its structural, chemical, and textural features relevant to ammonium polishing under conditions representative of RAS. Emphasis was placed on attributes associated with mechanical robustness, surface chemistry, and pore structure relevant to potential fixed‑bed operation rather than achieving maximum adsorption capacity under idealized laboratory conditions.

**Fig. 1:**
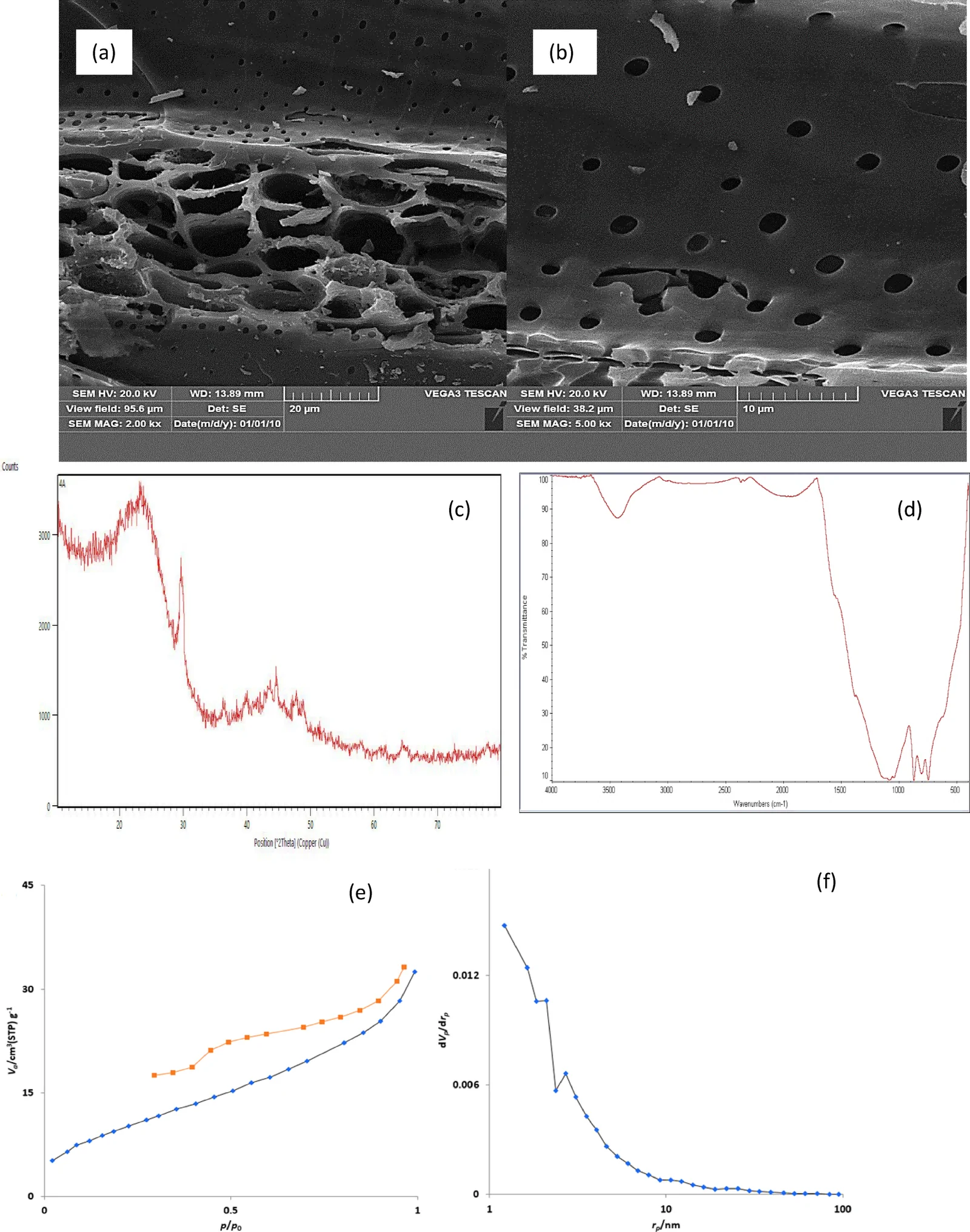
Structural characterization of *PS* biochar produced at 500 °C. (**a**,**b**) SEM micrographs at different magnifications; (**c**) X‑ray diffraction (XRD) pattern displaying broad peaks around 2θ = 23° and 43°, (**d**) Fourier transform infrared (FTIR) spectrum (**e**) Nitrogen adsorption–desorption isotherm (Type IV, H_3_ hysteresis) and (**f**) BJH pore size distribution curve confirming predominance of mesopores.

Scanning electron microscopy (SEM) images (Fig. 1a–b) revealed a heterogeneous and highly porous surface morphology characterized by irregular, slit‑shaped pores and fibrous channels distributed throughout the carbon matrix. Interconnected voids were observed across multiple length scales, indicating the coexistence of meso‑ and macroporous structures. Although partial collapse of plant tissues occurred during pyrolysis, the layered carbon framework remained largely intact, preserving an open structure that allows fluid penetration through the coarse biochar particles. The observed morphology is consistent with slit‑type pore systems typically associated with aggregated carbon platelets^50^.

The crystalline structure of the biochar was examined using X‑ray diffraction (XRD) analysis. As shown in Fig. 1c, two broad diffraction features centered at approximately 2θ ≈ 23° and 43° were observed, corresponding to the (002) and (100) planes of turbostratic carbon, respectively. The absence of sharp crystalline reflections confirms the predominantly amorphous nature of the biochar and indicates a low degree of graphitization. Such disordered carbon structures are characteristic of lignocellulosic biochars produced at moderate pyrolysis temperatures and are associated with heterogeneous surface energy distributions^51^.

Surface functional groups were identified by FTIR spectroscopy (Fig. 1d). A broad absorption band centered at 3434 cm^−1^ was assigned to O–H stretching vibrations of hydroxyl and phenolic groups. The peak observed at 1926 cm^−1^ corresponds to C = O stretching vibrations of carbonyl or conjugated functional groups. Additional bands at 1083 cm^−1^ were attributed to C–O stretching vibrations of alcohol and ether groups. Furthermore, peaks at 871 and 747 cm^−1^ were associated with out‑of‑plane bending vibrations of aromatic C–H bonds. Overall, the FTIR spectrum confirms the presence of multiple oxygen‑containing functional groups on the biochar surface^52^.

Textural properties were evaluated using N_2_ adsorption–desorption analysis at 77 K. The adsorption isotherm exhibited Type IV behavior with an H_3_ hysteresis loop (Fig. 1e), indicating a mesoporous structure dominated by slit‑shaped pores formed by aggregated carbon layers^25^. BET analysis yielded a specific surface area of 37.36 m^2^ g^−1^, an average pore diameter of 29.3 nm, and a total pore volume of 0.0499 cm^3^ g^−1^. The corresponding BJH pore size distribution (Fig. 1f) confirmed that the majority of pores were within the mesoporous range (10–50 nm). This pore structure is characteristic of coarse‑grained biochars produced by slow pyrolysis.

Electrostatic surface properties were assessed through zeta potential measurements conducted at pH 7.0. The biochar exhibited a slightly negative surface charge of − 0.7 mV, with electrophoretic mobility values centered near neutrality. This near‑neutral surface charge suggests the dominance of weakly dissociating oxygenated functional groups rather than strongly acidic or basic sites, which is consistent with operating conditions commonly encountered in biologically active aqueous systems^53^.

Elemental composition determined by CHNS analysis confirmed a carbon‑rich material, with carbon, hydrogen, and nitrogen contents of 76.2%, 2.9%, and 0.6%, respectively, while sulfur content was below 0.2%. The high carbon content and low heteroatom fractions indicate a chemically stable and non‑toxic adsorbent matrix suitable for repeated exposure to aquaculture effluents^54^. Collectively, these physicochemical characteristics describe *PS* biochar as a mechanically stable, mesoporous, and chemically active material with properties aligned toward practical adsorption applications rather than purely high‑capacity batch performance.

FTIR spectra before and after ammonium adsorption showed no discernible peak shifts or loss of characteristic functional groups. XRD patterns exhibited no detectable changes in crystalline phases or structural ordering following adsorption. SEM images confirmed preservation of particle morphology and pore architecture after exposure to ammonium‑containing solutions.

### Influence of operational parameters on nitrogen removal

The influence of key operational parameters, including contact time, adsorbent dosage, and initial solute concentration, on the removal efficiencies of ammonium (NH_4_^+^), nitrite (NO_2_^−^), and nitrate (NO_3_^−^) by coarse PS biochar was systematically evaluated under batch conditions. The experiments were designed to reflect concentration ranges and operating conditions relevant to post‑nitrification polishing scenarios in recirculating aquaculture systems.

#### Effect of contact time

**Fig. 2:**
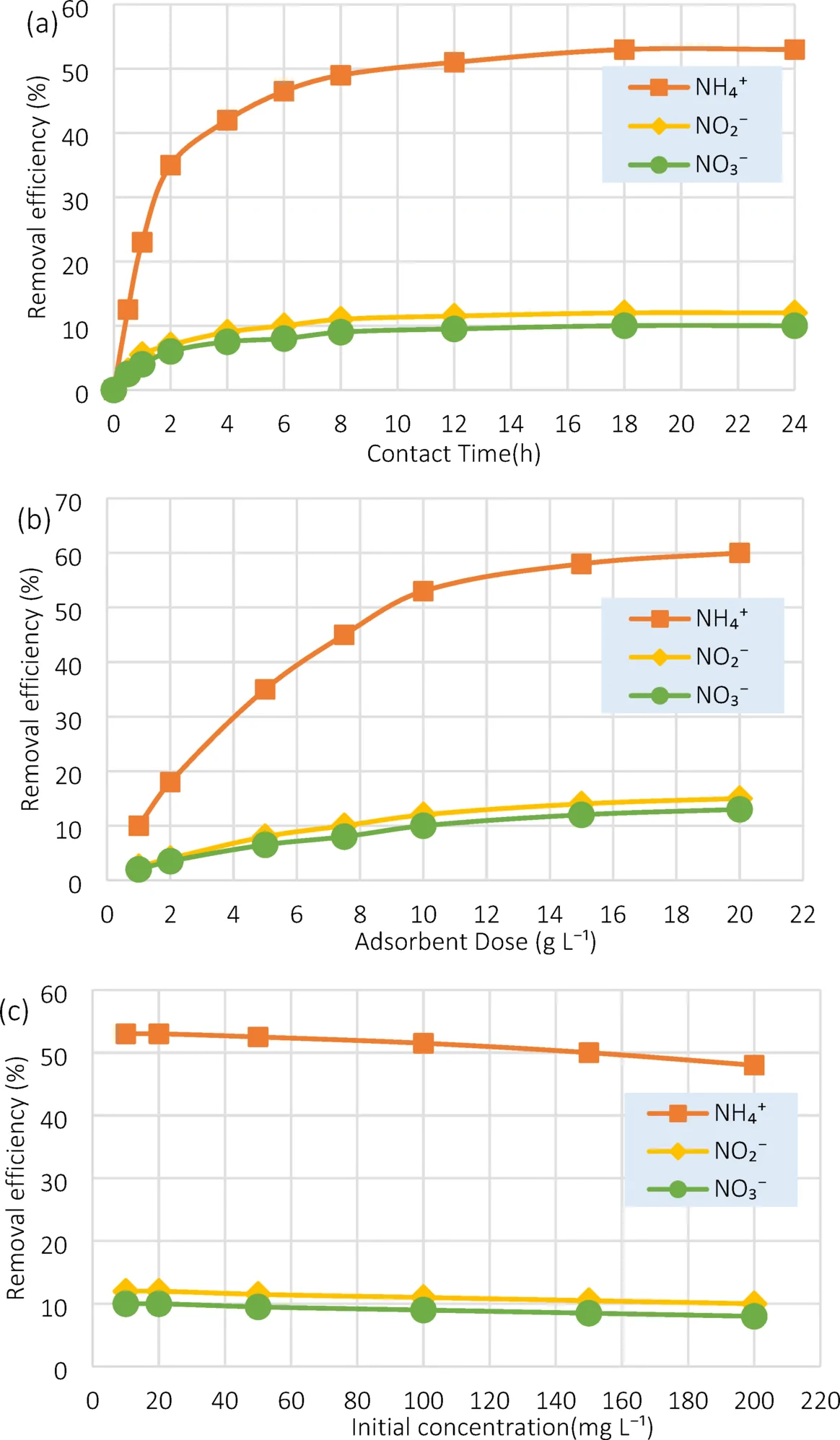
Effect of operational parameters on nitrogen removal performance by coarse *PS* biochar (2–4 mm). (a) Effect of contact time on the removal efficiency of NH_4_^+^​, NO_2_^−^​, and NO_3_^−^​ ​ (C_0_ = 50 mg L^−1^, dose = 10 g L^−1^, T = 25 °C).(b) Effect of adsorbent dose (contact time = 24 h).(c) Effect of initial concentration (dose = 10 g L^−1^, T = 25 °C).

The effect of contact time on nitrogen removal efficiency is presented in Fig. 2a. For all three nitrogen species, adsorption followed a biphasic trend characterized by a rapid initial uptake phase followed by a slower approach to equilibrium^55^. Ammonium exhibited the highest removal efficiency among the tested species. Approximately 40% of total NH_4_^+^ removal occurred within the first 4 h, after which the adsorption rate progressively decreased, reaching an apparent equilibrium after approximately 18 h. The maximum NH_4_^+^ removal efficiency achieved after 24 h was 53%.

In contrast, NO_2_^−^ and NO_3_^−^ exhibited substantially lower removal efficiencies throughout the contact time range. Equilibrium for both anions was attained within 12–18 h, with maximum removal efficiencies of approximately 12% for NO_2_^−^ and 10% for NO_3_^−^ at 24 h. The markedly lower uptake of nitrite and nitrate relative to ammonium indicates a strong selectivity of the biochar toward cationic nitrogen species under near‑neutral pH conditions.

#### Effect of adsorbent dosage

The influence of biochar dosage on nitrogen removal efficiency is shown in Fig. 2b, with adsorbent doses varied from 1 to 20 g L^−1^ at a fixed contact time of 24 h. Increasing the adsorbent dose resulted in a proportional rise in removal efficiencies for all three nitrogen species, reflecting the progressive availability of adsorption sites.

For NH_4_^+^, removal efficiency increased sharply from approximately 10% at a dosage of 1 g L^−1^ to 53% at 10 g L^−1^. Beyond this dosage, further increases in biochar concentration yielded only marginal improvements, with a maximum NH_4_^+^ removal of approximately 58% observed at 20 g L^−1^. A similar saturation trend was observed for NO_2_^−^ and NO_3_^−^, whose removal efficiencies stabilized at approximately 14% and 12%, respectively, at the highest adsorbent dosages tested.

The diminishing gains in removal efficiency at dosages above 10 g L^−1^ indicate that adsorption site availability was no longer the limiting factor under these conditions, and that equilibrium was governed by solute–surface interactions rather than adsorbent mass.

#### Effect of initial nitrogen concentration

Figure 2c illustrates the effect of initial nitrogen concentration, ranging from 10 to 200 mg L^−1^, on removal efficiency at a fixed biochar dosage of 10 g L^−1^ and a constant temperature of 25 °C. Across the tested concentration ranges of 10–100 mg L^−1^, the removal efficiency of NH_4_^+^ remained relatively stable and was maintained near 53%. At higher initial concentrations, a gradual decline in removal efficiency was observed, reaching approximately 48% at an initial concentration of 200 mg L^−1^.

The removal efficiencies of NO_2_^−^ and NO_3_^−^ remained consistently low across the entire concentration range, fluctuating narrowly between 10% and 12% and showing no pronounced dependency on initial concentration. The observed stability of removal efficiency with increasing solute concentration suggests that adsorption performance within the investigated range was not strongly constrained by mass‑transfer limitations under batch conditions.

### Adsorption kinetics of ammonium

The adsorption kinetics of ammonium (NH_4_^+^) onto PS biochar were investigated to elucidate the rate‑controlling steps and the underlying adsorption mechanisms under batch conditions. Kinetic experiments were conducted at an initial nitrogen concentration of 50 mg L^−1^, a biochar dose of 10 g L^−1^, and a temperature of 25 °C. The experimental data were analyzed using three widely applied kinetic models, including the pseudo‑first‑order, pseudo‑second‑order, and intraparticle diffusion models.

Owing to the substantially higher uptake capacity and removal efficiency of NH_4_^+^ compared with nitrite and nitrate under identical experimental conditions, detailed kinetic modeling and graphical interpretation were primarily focused on ammonium. In contrast, the adsorption of NO_2_^−^ and NO_3_^−^ remained very limited (< 13%), resulting in low equilibrium adsorption capacities and weakly developed kinetic profiles, which limit the reliability of detailed mechanistic interpretation based on kinetic model fitting. Nevertheless, for completeness, the kinetic parameters of NO_2_^−^ and NO_3_^−^ are reported numerically in Table 2. According to the results, pseudo‑first‑order model exhibited a moderate correlation with the experimental ammonium adsorption data (R^2^ = 0.971); In contrast, the pseudo-second-order model provided an excellent fit to the experimental data, with a high correlation coefficient (R^2^ = 0.995). The equilibrium adsorption capacity predicted by the model was in close agreement with the experimentally determined value.

**Fig. 3:**
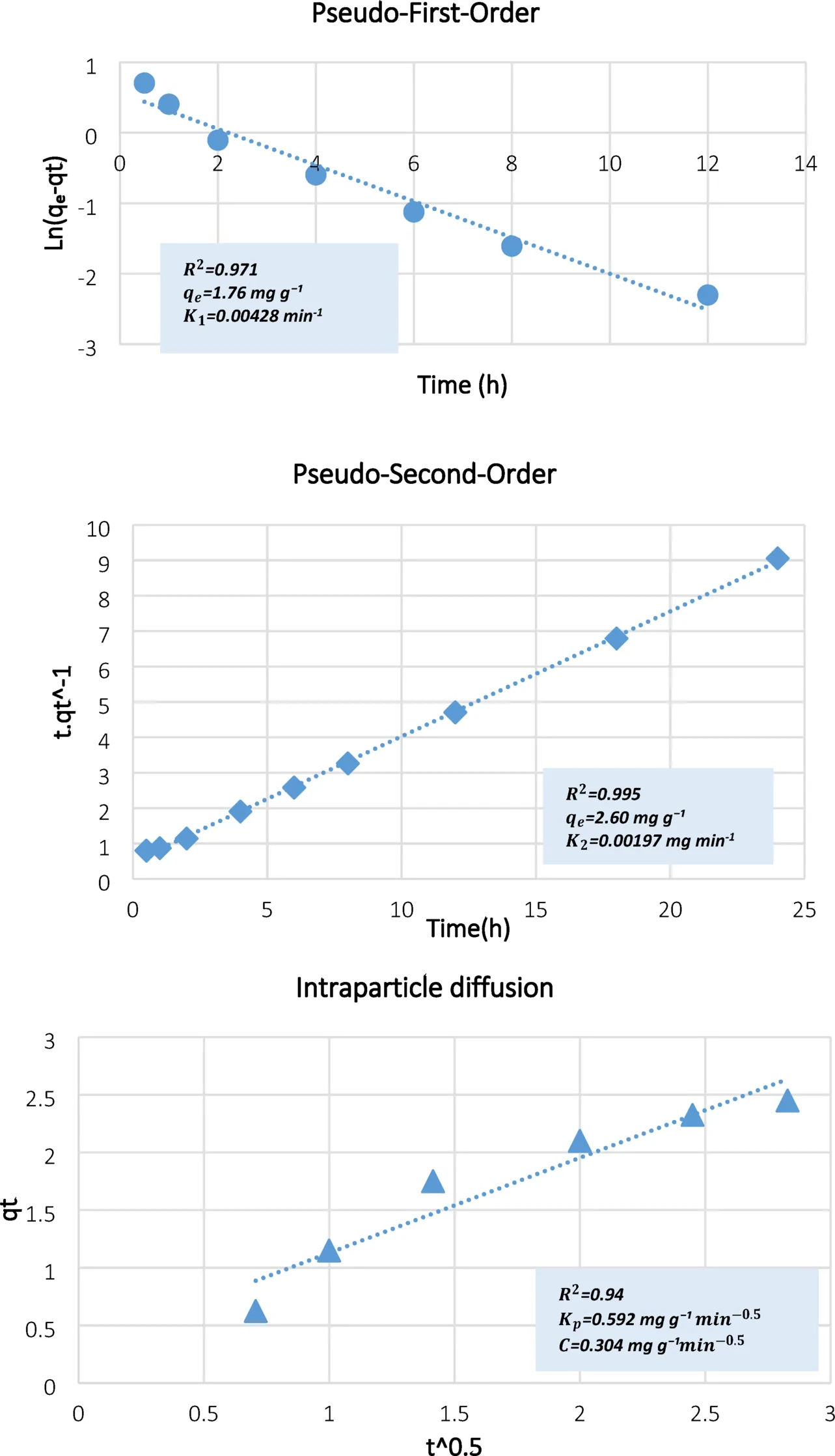
Kinetic model fitting plots for NH_4_^+^adsorption onto *PS* biochar (temperature = 25 °C, dose = 10 g L^−1^, C_0_ = 50 mg L^−1^); (**a**) Pseudo-first-order model (**b**) Pseudo-second-order model, and (**c**) Intraparticle diffusion model.

Further insight into the adsorption mechanism was obtained from the intraparticle diffusion analysis. The intraparticle diffusion plots for ammonium adsorption displayed a multilinear trend, indicating that the uptake process proceeded through multiple sequential stages. The initial linear segment corresponds to rapid external mass transfer and boundary‑layer diffusion, while the subsequent linear region reflects intraparticle diffusion of NH_4_^+^ ions into the internal pore structure of the biochar^56^. Notably, the intraparticle diffusion plots did not pass through the origin (C ≠ 0), confirming that intraparticle diffusion was not the sole rate‑limiting step and that external mass‑transfer resistance also contributed to the overall adsorption kinetics^57^ (Fig. 3).

**Table 2:** Kinetic parameters for nitrogen adsorption by coarse *Prunus spinosa* biochar*.

Model	Parameter	NH_4_^+^	NO_2_^−^	NO_3_^−^
Experimental	q_e_ (mg g^−1^)	2.65	0.60	0.50
Pseudo-first-order	q_e_,_cal_ (mg g^−1^)	1.76	0.41	0.36
	k_1_ (min^−1^)	0.00428	0.00408	0.00388
	R^2^	0.971	0.987	0.983
Pseudo-second-order	q_e_ (mg g^−1^)	2.60	0.64	0.49
	k_2_ (×10^−3^ g mg^−1^ min^−1^)	1.97	3.00	2.00
	h = k_2_ q_e_^2^ (mg g^−1^ min^−1^)	0.0133	0.00123	0.00048
	R^2^	0.995	0.99	0.998
Intraparticle diffusion	kp (mg g^−1^h^−0.5^)	0.592	0.1765	0.1584
	C (mg g^−1^)	0.304	0.0733	0.0401
	R^2^	0.94	0.9577	0.9421

*Experimental conditions: temperature = 25 °C, dose = 10 gL^− 1^, C_0_ = 50 mgL^− 1^.

### Equilibrium isotherm behavior

**Fig. 4:**
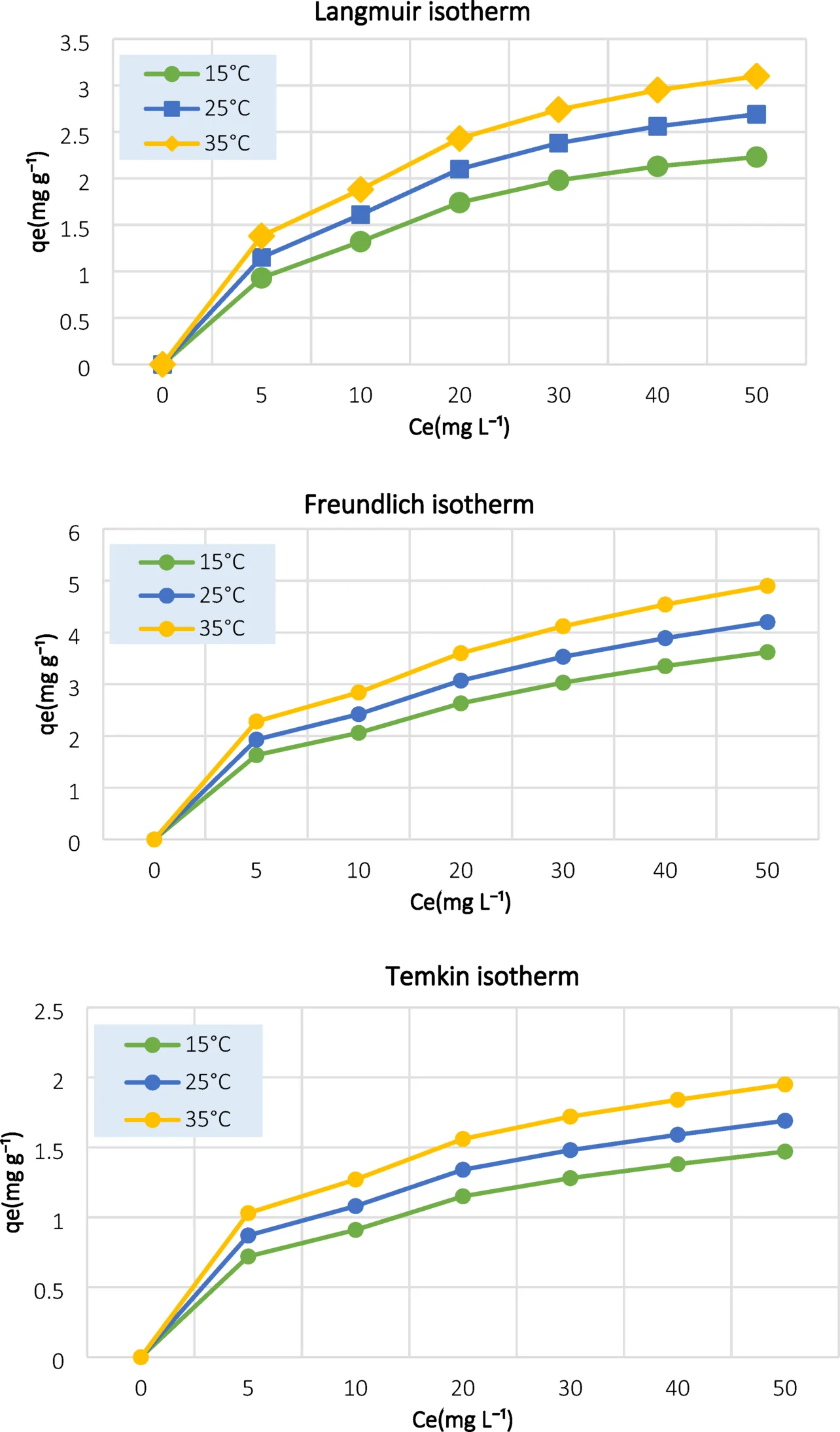
Linearized adsorption isotherms of NH_4_^+^ removal by PS biochar at different temperatures (dose = 10 g L^−1^, C_0_ = 50 mg L^−1^): (**a**) Langmuir model, (**b**) Freundlich model, and (**c**) Temkin model.

The equilibrium adsorption behavior of ammonium onto Prunus spinosa biochar was investigated at different temperatures (15, 25, and 35 °C) under batch equilibrium conditions to describe the interaction between ammonium ions and the biochar surface. The experimental data were analyzed using the Langmuir, Freundlich, and Temkin isotherm models. Linearized plots of the three models are presented in Fig. 4, and the corresponding parameters are summarized in Table 3.

The Langmuir model provided the best fit to the experimental data at all studied temperatures, as reflected by the highest coefficients of determination (R^2^ = 0.987 at 15 °C). The maximum monolayer adsorption capacity (q_m_) increased slightly with temperature, from 3.24 mg g^−1^ at 15 °C to 3.57 mg g^−1^ at 35 °C.

Similarly, the Freundlich model described the adsorption data satisfactorily, although with lower R^2^ values compared with the Langmuir model. The Freundlich constants (*n* ≈ 2.1–2.3) remained greater than unity across the investigated temperature range, confirming favorable adsorption, while 1/*n* < 1 indicates moderate surface heterogeneity typical of biochars derived from lignocellulosic biomass^58^. In comparison, the Temkin model yielded the lowest coefficients of determination among the three evaluated isotherms, indicating a comparatively poorer fit to the experimental equilibrium data.

### Thermodynamic parameters and thermal stability

**Fig. 5:**
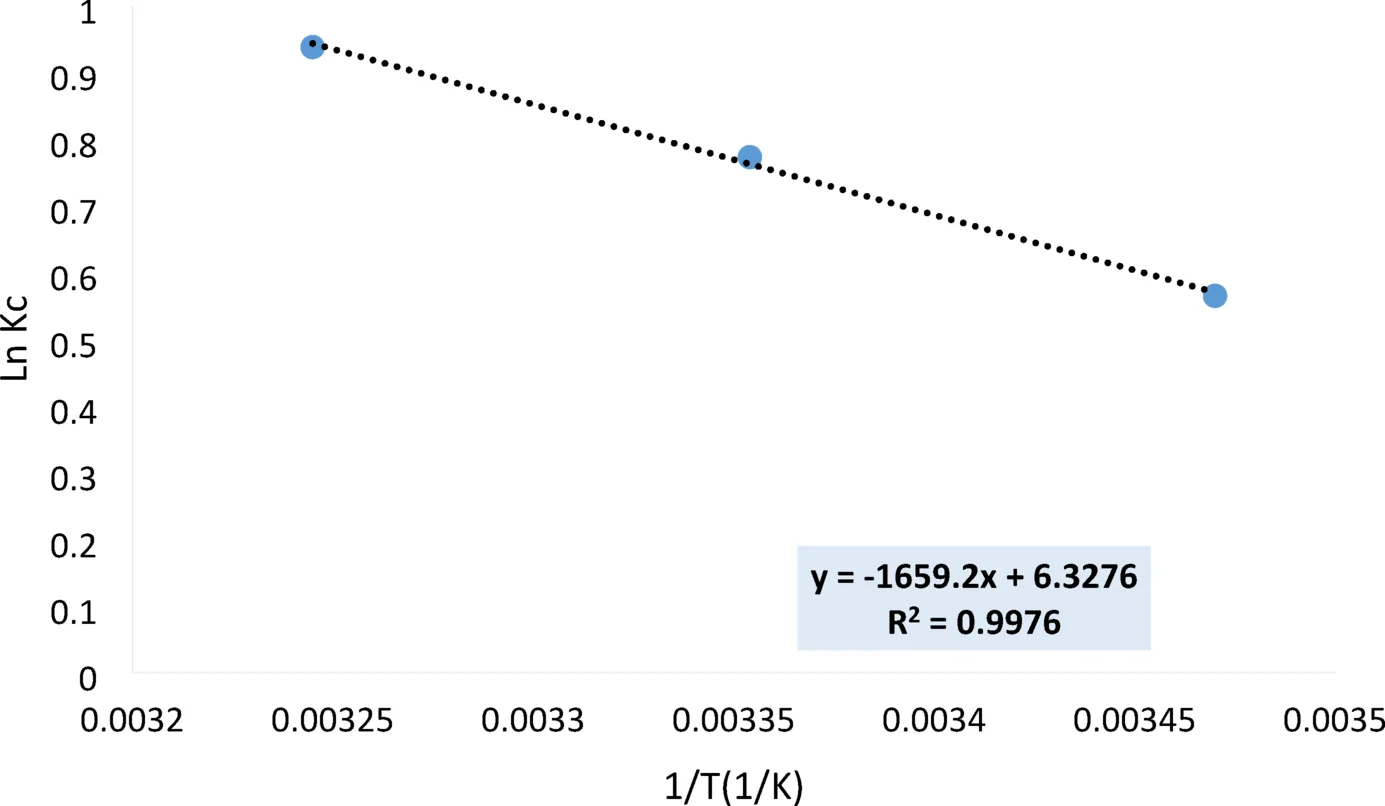
Van’t Hoff plot for the adsorption of ammonium ions onto *Prunus spinosa* biochar at different temperature.

The thermodynamic parameters for ammonium adsorption onto PS biochar were determined using equilibrium constants (K_c_​) derived from the Langmuir isotherms at 15, 25, and 35 °C. The van’t Hoff plot, illustrating the relationship between lnK_c_ and 1/T, is presented in Fig. 5. A linear regression was obtained across the studied temperature range with a correlation coefficient (R^2^) of 0.9976. From the slope and intercept of the regression equation, ΔH^0^ and ΔS^0^ were calculated as + 13.79 kJ mol^− 1^ and + 52.61 J mol^− 1^ K^− 1^1, respectively. ΔG^0^ values were − 1.37 kJ mol^−1^at 15 °C, − 1.89 kJ mol^− 1^ at 25 °C, and − 2.42 kJ mol^− 1^ at 35 °C. An increase in temperature from 15 to 35 °C resulted in a progressive increase in the equilibrium constant (K_c_​) and a corresponding increase in the negative magnitude of ΔG^0^.

**Table 3:** Isotherm parameters for the adsorption of NH_4_^+^ onto *Prunus spinosa* biochar at different temperatures (dose = 10 g L^−1^, C_0_ = 50 mg L^−1^).

Model	Parameter	Unit	15 °C	25 °C	35 °C
Langmuir	q_m_	mg g^−1^	3.24	3.41	3.57
	K_L_	L mg^−1^	0.041	0.052	0.063
	R_L_​	–	0.32	0.28	0.25
	R^2^	–	0.982	0.987	0.984
Freundlich	K_F_​	mg^1-1/n^ L^1/n^ g^−1^	0.67	0.81	0.95
	1/n	–	0.47	0.44	0.43
	n	–	2.13	2.27	2.33
	R^2^	–	0.951	0.962	0.956
Temkin	A	L g^−1^	0.39	0.43	0.46
	B	J mol^−1^	0.47	0.51	0.54
	R^2^	–	0.936	0.944	0.939

### Structural robustness and applicability to fixed‑bed operation

The structural robustness and suitability of PS biochar for potential fixed‑bed ammonium polishing were evaluated based on intrinsic physicochemical properties, particle size selection, and adsorption behavior under aquaculture‑relevant operating conditions. Given that the present work was intentionally conducted as a batch‑based investigation, the assessment emphasizes engineering‑relevant indicators of material integrity rather than direct post‑adsorption structural characterization[59].

The biochar consisted of a deliberately selected coarse particle fraction, which is widely associated with enhanced mechanical resistance, improved hydraulic compatibility, and reduced risk of particle attrition in packed‑bed systems^60^. SEM imaging and textural analysis (Sect. 3.1) revealed an open porous architecture dominated by mesopores and macropores, facilitating solute accessibility while limiting excessive pressure losses that commonly occur with fine powdered sorbents^61^. Such structural features are particularly advantageous in downstream polishing units integrated into recirculating aquaculture systems, where continuous flow stability and low maintenance requirements are critical.

Nitrogen adsorption–desorption analysis indicated moderate specific surface area and pore volume values characteristic of slow‑pyrolyzed, structurally stable lignocellulosic biochars. While these characteristics inherently constrain maximum equilibrium adsorption capacity, they simultaneously contribute to higher physical durability and resistance to pore collapse or blockage under operational conditions^62^. This balance reflects a deliberate engineering trade‑off favoring structural integrity and process reliability over capacity maximization, consistent with the intended role of the biochar as a polishing medium rather than a primary nitrogen removal material.

Surface chemical analysis further supports the inferred stability of the biochar matrix. FTIR and XRD results indicated predominance of stable aromatic carbon domains with limited crystalline mineral phases, while zeta potential measurements near neutral pH suggest weak electrostatic interactions under the tested conditions. This combination of features reduces the likelihood of excessive interparticle aggregation, fines generation, or chemical degradation when exposed to biologically treated aquaculture effluents containing diverse background ions.

Importantly, the ammonium concentrations investigated in this study correspond to low‑strength post‑nitrification polishing conditions, where the total adsorbed mass loading remains limited. Under such regimes, substantial alteration of pore structure or surface chemistry of coarse biochar materials is not generally expected^63^. Accordingly, the evaluation of structural integrity in the present work is based on intrinsic material characteristics and adsorption behavior rather than on pronounced post‑adsorption transformations.

From a process performance perspective, the consistent adsorption behavior observed across the investigated temperature range (15–35 °C) suggests resilience of the biochar under thermal conditions representative of operational variability in RAS. The absence of abrupt performance deterioration or anomalous experimental behavior further supports the mechanical and functional suitability of the material for preliminary fixed‑bed consideration, although continuous‑flow validation remains a necessary step for full‑scale implementation.

Overall, these findings indicate that coarse PS biochar possesses sufficient structural robustness and physicochemical stability to support its use as a downstream ammonium polishing medium in fixed‑bed configurations. While additional studies involving cyclic loading, regenerat, and column operation are required to fully quantify long‑term durability, the present results establish a defensible engineering basis for translating batch‑derived insights into practical polishing system design.

#### Morphological and textural stability

Post‑adsorption scanning electron microscopy (SEM) images revealed no visible signs of particle fragmentation, pore collapse, or structural degradation following ammonium uptake. The characteristic slit‑shaped pores and fibrous channels observed in the pristine biochar were largely preserved after adsorption, indicating that interaction with ammonium ions did not compromise the physical framework of the carbon matrix.

The absence of noticeable surface erosion or micro‑cracking confirms that the coarse biochar fraction retained its mechanical robustness throughout the adsorption process. This stability is particularly relevant for downstream polishing applications, where repeated hydraulic loading and prolonged contact with treated effluent are expected.

#### Chemical integrity and surface functionality

FTIR spectra collected before and after ammonium adsorption displayed comparable peak positions and relative intensities, suggesting that the fundamental surface chemistry of the biochar remained intact. The major absorption bands associated with hydroxyl (O–H), carbonyl (C = O), and ether/alcohol (C–O) functional groups showed no significant shifts or disappearances following adsorption.

Minor variations in peak intensity were observed in the region corresponding to oxygen‑containing functional groups, which can be attributed to interactions between ammonium ions and surface‑accessible functional sites. Importantly, the absence of new absorption peaks indicates that no irreversible chemical transformation or surface degradation occurred during adsorption.

#### Implications for operational stability

The retention of both morphological structure and surface functional integrity after ammonium adsorption suggests that PS biochar exhibits a high degree of physicochemical stability under the investigated conditions. This stability complements the moderate adsorption energies inferred from thermodynamic analysis (Sect. 3.5), reinforcing the conclusion that ammonium uptake proceeds through reversible interactions rather than permanent structural modification^64^.

Collectively, these findings indicate that the biochar can sustain ammonium uptake without compromising its structural or chemical integrity, supporting its suitability for repeated or long‑term use in polishing applications where stability and material durability are prioritized over maximum adsorption capacity.

### Fixed‑bed column performance under post‑nitrification conditions

The continuous-flow performance of coarse PS biochar was evaluated using an up-flow fixed-bed column operated under conditions representative of post-nitrification effluents. The column experiment was designed as a complementary engineering assessment rather than a full breakthrough study.

Effluent sampling was initiated after approximately one hydraulic retention time (HRT ≈ 12.5 h). During column operation, the effluent ammonium concentration exhibited a gradual increase toward influent levels. Partial ammonium removal was observed during the initial operational period, with normalized effluent concentrations (C/C_0_) remaining below unity within the first 24–48 h of operation. Beyond this period, the effluent ammonium concentration approached the influent concentration, indicating the attainment of a quasi-steady exhausted state.

In contrast, nitrite and nitrate concentrations in the column effluent remained close to their respective influent levels throughout the monitored operation. No systematic removal of NO_2_^−^ or NO_3_^−^ was detected during the experimental period. Extended column operation up to 144 h revealed no evidence of delayed breakthrough behavior, secondary removal phases, or concentration rebound phenomena. Effluent concentration profiles beyond the early and intermediate operational periods exhibited no further systematic temporal variation.

From a hydraulic perspective, the column operated stably throughout the entire experimental duration. No observable pressure buildup, flow channeling, bed compaction, or particle washout was detected. The absence of clogging or headloss issues was maintained during prolonged continuous operation.

Overall, the fixed-bed column exhibited partial ammonium removal during the early stages of operation, negligible removal of nitrite and nitrate, and stable hydraulic performance under continuous-flow conditions.

### Regeneration and reuse performance under cyclic operation

The reusability of PS biochar was evaluated through multiple consecutive adsorption–regeneration cycles to assess its cyclic stability and operational durability. Regeneration was conducted using a mild NaCl solution (0.1 M) to promote ammonium desorption under gentle ionic conditions without inducing physical or chemical degradation.

 Figure 5 presents the ammonium removal efficiency of the biochar over successive regeneration cycles. The regenerated biochar retained a high fraction of its initial adsorption performance across five cycles, with only a marginal decline in removal efficiency. The regeneration efficiency decreased gradually from approximately 100% in the first cycle to 96.1% after the fifth cycle.

No visible structural degradation, particle fragmentation, or fines generation was observed during repeated regeneration and reuse. The biochar maintained its physical integrity and handling properties throughout the cyclic tests.

## Discussion

### Role of coarse biochar in post‑nitrification ammonium polishing

The present study positions coarse-grained PS biochar as a supplementary polishing medium for residual ammonium removal downstream of biological nitrification in recirculating aquaculture systems (RAS). In contrast to adsorption studies that prioritize high influent concentrations or maximum equilibrium capacities, the conceptual framework adopted here emphasizes operational stability under conditions characteristic of mature nitrifying units, where residual ammonium fluctuations rather than bulk nitrogen loads represent the dominant management challenge^65^. Within this context, the observed nitrogen uptake behavior supports the interpretation of PS biochar as a functionally selective polishing material rather than a broad-spectrum adsorbent. The preferential interaction with ammonium, coupled with limited affinity toward nitrite and nitrate, aligns with surface chemical characteristics typical of mildly oxidized biochars, including a slightly negative surface charge and the predominance of weakly dissociated oxygen-containing functional groups^66^. Under near-neutral pH conditions, these features are expected to favor cation association while offering limited electrostatic driving force for anion retention, as widely reported for lignocellulosic biochars with comparable surface chemistries^67^.

From a process engineering standpoint, this selectivity carries practical significance for post-nitrification treatment trains. In biologically stabilized RAS, nitrate generally exhibits low acute toxicity, whereas residual ammonium remains the principal driver of short-term toxicity risks and operational instability^68^. A polishing medium that selectively attenuates ammonium without perturbing downstream nitrate levels therefore complements, rather than interferes with, established nitrification performance. In this, PS biochar functions as a targeted buffer against transient ammonium excursions, such as those arising from feed loading shocks, temperature variations, or partial biofilter underperformance, rather than as a primary nitrogen removal technology.

Importantly, the preservation of this selective behavior under continuous-flow conditions further reinforces the suitability of coarse PS biochar for downstream polishing applications. Rather than maximizing sorptive capacity, the material’s role is best understood as providing a hydraulically compatible, chemically stable, and functionally specific polishing step that integrates seamlessly with biological nitrogen removal processes.

### Trade‑off between adsorption capacity and engineering feasibility

The adsorption performance of PS biochar should be interpreted within the context of an intentional engineering trade-off rather than as a material shortcoming. In fixed-bed polishing applications, particularly under post-nitrification conditions, design priorities extend beyond maximizing equilibrium uptake to include hydraulic stability, mechanical durability, and predictable long-term operation under continuous flow. From this perspective, the use of coarse-grained biochar represents a strategic compromise that favors process reliability over absolute adsorption capacity^69^.

Coarse particle size inherently limits specific surface area relative to finely milled or chemically activated biochars; however, this reduction does not preclude functional adsorption performance when evaluated against polishing-oriented objectives. Mesoporous structures characteristic of lignocellulosic biochars remain accessible to ammonium ions at low residual concentrations, while simultaneously promoting favorable permeability and reducing the risk of excessive head loss or bed compaction. Such attributes are widely recognized as critical prerequisites for stable fixed-bed operation in water treatment systems^70,71^. Within the operational context of RAS, where polishing units are expected to buffer transient ammonium excursions rather than serve as primary removal technologies, hydraulic compatibility and mechanical integrity often govern system viability more strongly than theoretical capacity metrics^72^. Materials optimized exclusively for high surface area frequently require fine particle sizes or aggressive activation, which can compromise bed stability and limit practical scalability^73^. By contrast, the structural configuration of coarse PS biochar supports continuous-flow operation while maintaining sufficient sorptive functionality to fulfill its intended polishing role.

Accordingly, the performance profile of PS biochar aligns with the functional demands of post-nitrification polishing, in which robustness, operational resilience, and ease of integration outweigh the pursuit of maximum equilibrium adsorption capacity. This trade-off underscores the importance of evaluating adsorbent materials through an engineering lens that accounts for real-world operational constraints rather than capacity-driven benchmarks alone^74^.

### Kinetic and equilibrium insights relevant to polishing operation

The kinetic behavior of ammonium uptake by coarse PS biochar provides important insights into the nature of the governing interaction processes relevant to post-nitrification polishing. The conformity of experimental data to pseudo-second-order kinetics indicates that adsorption is predominantly controlled by surface-associated interactions rather than by a single diffusion-limited mechanism^75^. While pseudo-second-order behavior is frequently interpreted as evidence of chemisorption, model conformity alone does not constitute definitive proof of irreversible surface complexation or electron sharing. In the present system, the kinetic response is more appropriately interpreted as reflecting surface-controlled physicochemical interactions that remain largely reversible in nature^76^.

This interpretation is further supported by intraparticle diffusion analysis, which exhibits multi-linear behavior indicative of sequential uptake stages. The initial region reflects rapid external mass transfer, followed by a slower diffusion process within the internal pore structure of the biochar^77^. The systematic deviation of intraparticle diffusion plots from the origin demonstrates that intraparticle diffusion does not act as the sole rate-limiting step, confirming a mixed kinetic control regime. Such kinetic characteristics are particularly advantageous for post-nitrification polishing applications, where rapid attenuation of low-level residual ammonium must be coupled with sustained uptake under continuous operation, rather than short-term maximization of equilibrium capacity^78,79^.

Equilibrium adsorption behavior further reinforces this polishing-oriented interpretation. The strong conformity of ammonium uptake to the Langmuir isotherm suggests that adsorption proceeds predominantly via monolayer coverage on a finite number of energetically comparable surface sites^80^. Within the low concentration range characteristic of post-nitrification effluents, such behavior supports a stable and predictable uptake regime. From an engineering standpoint, this equilibrium profile favors gradual saturation rather than abrupt exhaustion, thereby facilitating predictable fixed-bed operation and reliable regeneration scheduling without sudden performance decline^48^.

The comparatively weaker fit of the Freundlich model indicates that surface heterogeneity and multilayer adsorption effects play a secondary role under the investigated conditions, while the poor performance of the Temkin model suggests that a progressive decrease in adsorption heat with increasing surface coverage is not the dominant governing mechanism. Collectively, the isotherm analysis demonstrates that ammonium adsorption onto PS biochar is best described by Langmuir-type behavior within the tested concentration and temperature ranges, consistent with adsorption occurring on energetically uniform and functionally accessible surface sites.

Taken together, the combined kinetic and equilibrium responses point toward a coherent physicochemical interaction framework dominated by surface-controlled, reversible processes. The preferential uptake of NH_4_^+^ is consistent with the presence of oxygen-containing surface functional groups and a slightly negative surface charge under circumneutral pH conditions, which collectively favor cation exchange with secondary electrostatic contributions. Similar interaction mechanisms have been widely reported for lignocellulosic biochars exhibiting comparable surface chemistries^81–83^.

This mechanistic interpretation is further corroborated by post-adsorption material characterization and regeneration behavior. The absence of detectable alterations in FTIR spectra, XRD patterns, and SEM morphology following ammonium exposure indicates that adsorption does not induce irreversible modification of surface chemistry, crystalline structure, or pore architecture. Such stability supports adsorption governed primarily by reversible physicochemical interactions rather than strong surface complexation or material degradation. The high regeneration efficiency achieved using a mild NaCl solution provides complementary evidence that ammonium binding occurs at energetically moderate sites characteristic of ion-exchange-type mechanisms, enabling effective desorption without structural stress or chemical alteration of the biochar matrix^84^.

Importantly, this reversible interaction framework aligns with the functional requirements of post-nitrification polishing, where repeated adsorption–regeneration cycles and long-term material integrity are essential. The preservation of oxygen-containing surface functionalities, including hydroxyl, carbonyl, and ether moieties, facilitates preferential interactions with monovalent ammonium ions while inherently limiting the uptake of anionic nitrogen species such as nitrite and nitrate. This selectivity directly supports the role of PS biochar as a targeted polishing medium rather than a broad-spectrum nitrogen adsorbent^85^. Thermodynamic trends exhibiting moderate driving forces further reinforce adsorption dominated by physical–chemical interactions rather than irreversible surface bonding^86^.

Within the context of continuous-flow systems, the batch-derived kinetic and equilibrium parameters presented here should be interpreted as an explanatory framework rather than as direct predictors of column breakthrough behavior. While equilibrium capacity defines an upper theoretical limit under idealized conditions, practical fixed-bed performance is constrained by hydraulic residence time, partial bed utilization, and gradual saturation dynamics^48^. Notably, the surface-controlled uptake kinetics inferred from pseudo-second-order modeling indicate that the characteristic adsorption time scale is compatible with typical empty bed contact times employed in polishing columns. This time-scale compatibility provides a mechanistic basis for the gradual and predictable breakthrough behavior expected under continuous operation, reinforcing the relevance of batch-derived insights for engineering interpretation rather than direct quantitative design^35,48,87^.

### Thermodynamic implications and temperature sensitivity

The thermodynamic behavior of ammonium adsorption onto PS biochar provides an important complementary perspective to the kinetic and equilibrium insights discussed in Sect. 4.3, particularly with respect to temperature sensitivity and operational robustness under realistic aquaculture conditions. The observed temperature-dependent trends indicate that ammonium uptake becomes increasingly favorable at elevated temperatures, a behavior that is consistent with enhanced adsorbate–surface interactions under higher thermal energy conditions. Such temperature responsiveness aligns with the isotherm-derived increase in Langmuir monolayer capacity (q_m_) and supports the interpretation that adsorption performance is maintained or slightly enhanced under warm-water aquaculture regimes^88,89^.

The moderate magnitudes of the thermodynamic parameters further clarify the nature of the governing interaction mechanisms. Specifically, the values of ΔG° and ΔH° fall within a range characteristic of reversible physical–chemical adsorption processes rather than strong, irreversible surface bonding^35^. From a mechanistic standpoint, this thermodynamic signature is consistent with adsorption dominated by surface-controlled interactions involving weak electrostatic forces and ion-exchange-type processes, rather than covalent bond formation or permanent surface transformation. This interpretation reinforces the kinetic and equilibrium analyses presented earlier, which collectively indicate adsorption governed by moderate interaction energies compatible with reversibility.

From an engineering perspective, such thermodynamic behavior is particularly advantageous for post-nitrification polishing applications. In contrast to strongly exothermic or highly temperature-sensitive chemisorption processes, the endothermic yet moderate interaction energies observed here support gradual capacity exhaustion and predictable saturation behavior. This enables controlled regeneration scheduling and reduces the risk of abrupt performance loss during prolonged operation. The absence of detectable thermal instability across the investigated temperature range (15–35 °C) further demonstrates compatibility with seasonal temperature fluctuations commonly encountered in recirculating aquaculture systems (RAS), where influent temperatures may vary substantially throughout the year^90,91^.

Importantly, the reversible nature of ammonium adsorption inferred from thermodynamic analysis is independently corroborated by regeneration performance. The high recovery efficiencies achieved using a mild NaCl solution indicate that ammonium uptake does not induce irreversible chemical modification or structural degradation of the biochar matrix^92^. This convergence of thermodynamic reversibility and practical regenerability provides a coherent and internally consistent framework linking molecular-scale interaction energetics to process-level operability.

It is also worth emphasizing that the relatively narrow range of thermodynamic parameters observed in this study should not be interpreted as a limitation. On the contrary, limited variation in ΔG°, ΔH°, and ΔS° across the 15–35 °C window reflects thermodynamic robustness within an engineering-relevant operating envelope. For post-nitrification polishing systems, such stability is desirable, as it implies predictable adsorption behavior under realistic and fluctuating environmental conditions rather than strong sensitivity to temperature perturbations. This contrasts with adsorption systems dominated by strongly temperature-dependent chemisorption, which may exhibit higher apparent driving forces but often suffer from reduced operational flexibility and limited regenerability^93,94^.

Taken together, the thermodynamic response of PS biochar aligns closely with the functional objectives of polishing-oriented treatment systems. The combination of moderate, endothermic, and reversible adsorption energetics supports sustained performance across typical aquaculture temperature ranges, facilitates repeated adsorption–regeneration cycles, and minimizes the risk of material degradation during long-term operation. These thermodynamic characteristics therefore reinforce the suitability of PS biochar as a temperature-tolerant and regenerable polishing sorbent, complementing the kinetic, equilibrium, and structural insights discussed in the preceding sections.

### Structural stability and relevance to fixed‑bed applicability

The preservation of structural integrity following ammonium exposure, as evidenced by post-adsorption morphological and spectroscopic assessments, constitutes a fundamental prerequisite for the transition of PS biochar from laboratory-scale batch experiments to continuous-flow fixed-bed applications. The absence of detectable morphological degradation or chemical alteration suggests that the adsorption process—previously characterized as a reversible physical–chemical interaction (Sect. 4.4)—proceeds without inducing significant internal strain or surface fouling within the biochar matrix. From a mechanistic standpoint, the retention of slit-shaped pores and fibrous channels is particularly significant, as these features govern internal mass transfer and ensure that surface sites remain accessible over repeated operational cycles. This physicochemical resilience addresses a critical concern in fixed-bed engineering, where the physical breakdown of media often leads to hydraulic channeling, increased headloss, or unpredictable breakthrough behavior^95,96^.

The findings from the short-term column assessment further substantiate the practical viability of using coarse-grained PS biochar in post-nitrification polishing stages. By maintaining selective ammonium uptake under continuous-flow conditions without compromising hydraulic function, the material demonstrates the intended engineering trade-off: prioritizing mechanical robustness and hydraulic conductivity over the maximized surface area typically offered by powdered or chemically fragile alternatives. In the context of RAS, where high flow rates and the potential for biofouling are inherent, the use of coarse particles (2–4 mm) serves as a strategic safeguard against clogging and pressure drop development. Such stability confirms that the observed adsorption characteristics are not merely transient phenomena but are supported by a material framework capable of sustaining performance under dynamic loading conditions^96–98^.

Furthermore, the consistency between the stable surface chemistry (FTIR) and the sustained hydraulic performance during continuous flow provides a coherent basis for the long-term reliability of the system. While the current study acknowledges that long-term durability under extended cyclic loading remains to be fully quantified, the integration of mechanical robustness with the moderate, reversible adsorption energetics discussed in Sect. 4.3 and 4.4 provides a defensible engineering foundation. Specifically, the lack of irreversible chemical modification ensures that the material can undergo multiple regeneration cycles—using the mild salt-based approach validated earlier—without the progressive loss of structural or functional capacity.

In conclusion, the structural stability of PS biochar reinforces its role as a practical and targeted polishing medium rather than a broad-spectrum nitrogen adsorbent. By aligning physicochemical durability with selective ammonium removal and favorable hydraulic characteristics, the material meets the stringent operational demands of full-scale RAS environments. This alignment between molecular-scale stability and process-scale reliability positions coarse-grained PS biochar as a viable technology for enhancing nitrogen management resilience in modern aquaculture systems, bridging the gap between fundamental adsorption science and applied engineering requirements.

### Implications for RAS process integration and future work

The holistic integration of the findings presented in this study suggests that coarse-grained PS biochar is most effectively utilized as a supplementary polishing medium rather than a standalone nitrogen removal technology. Its strategic role within a Recirculating Aquaculture System (RAS) is primarily centered on its capacity to dampen residual ammonium fluctuations—acting as a physicochemical buffer downstream of biological nitrification units. This buffering capacity is particularly critical during transient operational phases, such as feed-load shocks, sudden temperature shifts, or periods of biofilter instability, where biological kinetics may temporarily lag behind ammonium production rates^99^. By providing a rapid-response removal mechanism through the selective adsorption pathways discussed in Sect. 4.3, the biochar medium enhances the overall system resilience, ensuring that ammonium concentrations remain below toxicological thresholds for sensitive aquatic species.

The high level of cyclic stability and regeneration efficiency (as reported in Sect. 3.8) carries significant operational and economic implications. The minimal decline in removal performance over multiple cycles suggests that the active surface sites of PS biochar are not prone to irreversible poisoning or structural clogging under the low-load conditions typical of post-nitrification effluents. From a management perspective, this means that the adsorbent’s exhaustion is unlikely to be the primary driver of bed replacement. Instead, the effective service life in a full-scale RAS will likely be governed by hydraulic factors and external fouling processes. The ability to restore capacity using a mild, low-cost NaCl solution—coupled with the structural robustness of the coarse particles—directly supports the economic viability of the system, aligning with the screening-level cost estimate of approximately 0.30 USD m^−3^ and reinforcing the feasibility of its adoption in cost-sensitive aquaculture operations.

Despite these promising indicators, the transition from laboratory screening to pilot-scale implementation requires addressing several key uncertainties. Future research must prioritize long-term breakthrough studies under dynamic hydraulic loading to accurately quantify the volume-to-exhaustion ratios in real aquaculture water matrices. Furthermore, the potential for biofouling and the accumulation of recalcitrant organic matter on the biochar surface remain critical areas for investigation; these processes could alter the effective pore diameter and competitive adsorption dynamics over extended operation. Therefore, evaluating the interaction between the biochar’s physicochemical surface and the microbial ecology of the RAS effluent is essential. Addressing these parameters through long-term continuous-flow trials will provide the necessary engineering data to translate the demonstrated material suitability into robust, reliable, and scale-up-ready nitrogen management designs.

### Engineering trade‑offs governing applicability to fixed‑bed post‑nitrification polishing

 The comparative framework presented in Table 4 necessitates a fundamental paradigm shift in how adsorbent performance is evaluated for recirculating aquaculture systems. Unlike traditional adsorption studies that prioritize the maximization of equilibrium capacity (qm), an engineering-oriented investigation for post-nitrification polishing must operate within the constraints of realistic RAS environments. Within this context, Table 4 functions not as a competitive ranking of adsorption capacities, but as a strategic screening tool that illustrates the critical intersection between molecular-scale performance and process-scale feasibility.

A primary distinction highlighted by this comparison is the “concentration-capacity paradox.” While numerous engineered sorbents and activated biochars in the literature report ammonium capacities exceeding 10 mg g^−1^, these values are predominantly derived from high-strength solutions (often 100–500 mg L^−1^) that are decoupled from the operational reality of mature RAS. In post-nitrification effluents, where residual TAN typically fluctuates within a narrow regime of 2–5 mg L^−1^, the primary treatment objective is the stabilization of ammonium below chronic toxicity thresholds rather than the exhaustion of theoretical capacity^65^. Under such low-concentration conditions, the applicability of equilibrium-based metrics as the sole performance indicator is diminished. Instead, as synthesized in Table 4, factors such as hydraulic compatibility, structural robustness, and the energy footprint of production assume greater practical significance. The moderate capacity of PS biochar is, therefore, a deliberate design trade-off; by utilizing coarse-grained (2–4 mm) particles instead of finely powdered or chemically aggressive alternatives, the system prioritizes the prevention of pressure losses, particle washout, and clogging—challenges that frequently render high-capacity materials unsuitable for stable fixed-bed operation^100^.

This trade-off is further justified by the selectivity and regeneration dynamics discussed in the preceding sections. As indicated in Table 4, many high-performance materials require complex or costly regeneration strategies. In contrast, the ability of PS biochar to maintain its functional and structural integrity—confirmed by the post-adsorption SEM, FTIR, and XRD analyses—allows for a simplified regeneration protocol using a low-cost NaCl solution. This efficiency, which recovers over 80% of capacity, directly translates to the economic viability of the process, supporting a benchmarking treatment cost of approximately 0.30 USD m^−3^, as estimated in this study based on material production, regeneration efficiency, and operational lifespan considerations^73,101,102^. Furthermore, the preferential affinity of PS biochar for NH_4_^+^ over NO_2_^−^ and NO_3_^−^ minimizes the premature saturation of active sites by competing anions, effectively extending the service life of the filter bed despite its moderate absolute capacity.

The positioning of PS biochar within the “design niche” of polishing media reflects a balance between performance, robustness, and simplicity that is particularly advantageous for small- to medium-scale RAS facilities. While chemical modifications (e.g., KOH or HNO_3_ activation) could theoretically enhance the surface area of Prunus spinosa feedstock, such processes increase both material fragility and operational costs, often compromising the very mechanical integrity required for continuous-flow applications. By aligning the material’s physicochemical properties with the specific requirements of downstream nitrogen management, this study bridges the persistent gap between fundamental adsorption research and practical aquaculture engineering. Although long-term uncertainties such as biofouling accumulation under real biological loading remain to be quantified in future pilot-scale trials, the current engineering framework provides a defensible basis for the adoption of coarse biochar as a reliable and cost-effective safeguard for ammonium stabilization in modern RAS.

**Table 4:** Engineering trade‑offs of ammonium adsorbents reported in the literature with emphasis on fixed‑bed post‑nitrification polishing*.

Adsorbent	Target species	Particle form	Reported system	Performance indicator	Key engineering advantages	Main limitations	Reference
PS biochar	NH_4_^+^	Coarse (2–4 mm)	Batch (polishing-oriented)	q_m_=3.41 mg g^−1^ at C_0_ ≈ 50 mg L^−1^	Low-cost agro-waste material, hydraulic compatibility for packed beds, mechanically robust, easy regeneration, suitable for post-nitrification polishing	Moderate adsorption capacity compared with fine or chemically modified biochars	This study
Pine sawdust biochar (PS300)	NH_4_^+^	Not specified (reported as limitation)	Batch	q_m_=5.38 mg g^−1^ at C_0_ up to 100 mg L^−1^	Low cost, agro-waste derived, enhanced surface functionality at low pyrolysis temperature (300 °C), applicable to wastewater treatment	Particle size not reported; adsorption mainly governed by chemical bonding and electrostatic interactions	^103^
Wheat straw biochar (WS550)	NH_4_^+^	Not specified (reported as limitation)	Batch	q_m_ =2.08 mg g^−1^ at C_0_​ up to 100 mg L^−1^	Abundant agro-waste feedstock, potential reuse as nutrient-loaded soil amendment	Lower adsorption capacity than PS300 due to fewer surface functional groups; particle size not specified	^103^
Corncob-based modified biochar (MBCC)	NH_4_^+^	Coarse (0.5–2 mm)	Fixed-bed column	q_m_ =12.83 mg g^−1^ at C_0_​=10 mg L^−1^;	Tested under dynamic conditions, suitable for continuous-flow operation, improved binding sites via chemical modification, regenerable with NaOH/NaCl	Requires chemical modification; performance sensitive to influent concentration and flow rate; regeneration efficiency decreases over cycles	^104^
Modified corncob biochar (MBCC2)	NH_4_^+^	0.5–2 mm	Batch	q_m_ =22.6 mg g^−1^ at C_0_ = 10–100 mg L^−1^	High adsorption capacity due to HNO_3_/NaOH modification; good Langmuir/Sips fit; effective ammonium binding	Use of strong acids/bases; pH-dependent performance; batch configuration limits direct fixed-bed translation	^104^
Rice husk biochar (RHB)	NH_4_^+^, NO_2_^−^, NO_3_^−^	Coarse (5–8 mm)	Batch	q_m_(NH_4_^+^) = 0.1 mg g^−1^;(NO_2_^−^) = 0.2 mg g^−1^; (NO_3_^−^) = 0.15 mg g^−1^	Low-cost agro-waste material, hydraulic compatibility, applicable as biofilter media in RAS	Very low adsorption capacity due to large particle size; limited surface accessibility; reduced efficiency relative to finer biochars	^26^
Pomegranate peel powder (PPP)	NH_4_^+^	Powder (< 250 μm)	Batch	q_m_=6.18 mg g^−1^ at C_0_​=30 mg L^−1^	Very low-cost biosorbent, no chemical modification required, potential fertilizer reuse after adsorption	Powder form limits fixed-bed applicability; pH-sensitive (optimal pH ≈ 4); equilibrium influenced by mixing conditions	^105^
Bacterial cellulose activated carbon (BC-AC/10 M-500)	NH_4_^+^	Granular (0.5–1.0 mm)	Batch	q_m_ =221.4 mg g^−1^ at C_0_ =360 mg L^−1^	Extremely high capacity due to multi-porous structure and abundant oxygenated groups; renewable cellulose-based precursor	Requires KOH activation and high-temperature carbonization; evaluated at unrealistically high influent concentrations; not tested in continuous systems	^103^
Peanut husk powder (PH)	NH_4_^+^	Powder (63 μm)	Batch	q_m_ =1.454 mg g^−1^ at C_0_= 5 mg L^−1^;	Low-cost agro-waste, no modification required, reusable, environmentally benign	Low adsorption capacity; pH-dependent (optimal pH ≈ 7); batch and powder form limit scale-up	^106^
Modified peanut husk (mPH)	NH_4_^+^	Powder (63 μm)	Batch	q_m_ =2.15 mg g^−1^ at C_0_ = 5 mg L^−1^	High removal efficiency after NaOH/KMnO₄ modification; reusable for multiple cycles; effective on real water matrices	Chemical modification required; moderate adsorption capacity; pH-dependent performance	^106^
Peanut husk biochar (BPH)	NH_4_^+^	Powder (63 μm)	Batch	q_m_ =3.079 mg g^−1^ at C_0_ =5 mg L^−1^;	Produced at low pyrolysis temperature (300 °C); reusable; improved surface properties relative to raw husk	Lower efficiency than modified forms at low influent concentrations; requires pyrolysis; powder form limits fixed-bed use	^106^

* Note: Reported performances are synthesized as provided in the source studies and encompass divergent experimental objectives, particle sizes, system configurations, and influent regimes. Most literature values represent peak capacities achieved under batch conditions using fine or powdered adsorbents to maximize equilibrium uptake. In contrast, the present study emphasizes ammonium polishing using coarse-grained (2–4 mm) particles specifically selected for hydraulic stability and operational compatibility with fixed-bed RAS applications. Furthermore, while capacities reported in this study were derived under low equilibrium concentrations (Ce < 2 mg L^−1^) representative of post-nitrification conditions, many literature values were obtained at substantially higher influent concentrations (C_0_ > 50–100 mg L^−1^), which inherently enhance apparent maximum capacities. Therefore, direct comparisons should be interpreted within this engineering-oriented context rather than as a simple ranking of theoretical capacities.

### Economic feasibility and industrial scalability

The transition from fundamental adsorption kinetics to industrial RAS application necessitates a critical evaluation of economic viability. It must be explicitly emphasized that the economic assessment presented herein functions as a screening-level benchmarking estimate intended to establish engineering feasibility rather than a site-specific, high-fidelity techno-economic analysis. Within this framework, the preliminary analysis indicates that the proposed PS biochar system offers a substantial competitive advantage over conventional tertiary treatment technologies, with an estimated total treatment cost of approximately 0.30 USD m^−3^. This cost structure is underpinned by three synergistic engineering pillars:

(i) Low-enthalpy production and feedstock circularity: By utilizing locally sourced Prunus spinosa (PS) wood—a feedstock with negligible acquisition costs—and employing slow pyrolysis at a moderate temperature of 500 °C, the process significantly curtails the energy intensity and carbon footprint typically associated with high-temperature activation or chemical modification of carbons^107^.
(ii) Operational longevity and structural robustness: As discussed in Sect. 4.5 and 4.6, the mechanical integrity of the coarse particles minimizes attrition-driven material loss. This physical stability, combined with the material’s high regeneration efficiency, ensures that the initial sorbent investment is amortized over extended operational cycles, effectively reducing the frequency of bed replacement^108,109^.
(iii) Simplified regeneration protocols: The ability to recover over 80% of the adsorption capacity through a mild NaCl-based elution—retaining over 96% of its functional performance across multiple cycles—translates to a low-complexity maintenance regime that is particularly suited for the operational constraints of aquaculture facilities^92,110^.

When benchmarked against established nitrogen removal technologies, the PS biochar system demonstrates a clear “cost-performance niche.” Conventional ion-exchange processes and membrane filtration systems (e.g., UF/RO), which are frequently employed for ammonium polishing, incur operational costs ranging from 1.5 to 3.0 USD m^−3^ and 0.8–2.0 USD m^−3^, respectively^65,111,112^. These higher costs are often driven by expensive synthetic resins, high energy requirements for pressure-driven filtration, and the necessity for chemically aggressive regeneration agents. In contrast, the PS biochar system provides a “low-tech, high-reliability” alternative. For instance, in a representative medium-scale RAS facility (100 m^3^ day^−1^), the annual operating expenditure for biochar-based polishing would be significantly lower than that of ion exchange, reinforcing its suitability for cost-sensitive aquaculture paradigms where profit margins are closely tied to water treatment efficiency^113^.

Furthermore, the scalability of this system is supported by the simplicity of the fixed-bed configuration. Unlike biologically mediated nitrogen removal, which can be sensitive to sudden thermal or hydraulic shocks, the biochar polishing unit serves as a robust physicochemical buffer. While long-term industrial-scale performance remains to be validated through continuous-flow pilot trials, this screening-level economic framework provides a defensible basis for the adoption of coarse biochar as a scalable and economically sustainable safeguard for residual ammonium stabilization in modern closed-loop aquaculture systems.

## Conclusion

This study establishes a comprehensive engineering framework for the application of coarse-grained Prunus spinosa (PS) biochar as a selective ammonium polishing medium in recirculating aquaculture systems (RAS). By prioritizing hydraulic compatibility and mechanical robustness over the pursuit of maximum adsorption capacity—a common limitation in laboratory-scale biochar research—this work demonstrates that the 2–4 mm particle size fraction provides a strategic balance between sorptive functionality and fixed-bed operational reliability.

The core findings confirm that PS biochar functions as an effective physicochemical buffer, capable of selectively attenuating residual ammonium fluctuations downstream of biological nitrification units. The adsorption process is governed by reversible, surface-controlled interactions (consistent with pseudo-second-order kinetics and Langmuir isotherms) which preserve the material’s structural and chemical integrity over multiple operational cycles. Mechanistically, the preferential uptake of NH_4_^+^ is driven by ion-exchange and electrostatic interactions within the lignocellulosic matrix, while anionic nitrogen species (NO_2_^−^ and NO_3_^−^) remain largely unaffected, ensuring the preservation of nitrification efficiency.

From an engineering perspective, the deliberate trade-off between particle size and surface area is justified by the biochar’s superior hydraulic performance, characterized by the absence of clogging or excessive head loss—critical prerequisites for stable industrial-scale operation. Furthermore, the demonstrated regenerability of the material using a mild NaCl solution, combined with an estimated treatment cost of 0.30 USD m^−3^, positions PS biochar as a cost-effective and sustainable alternative to conventional ion-exchange resins and membrane-based nitrogen polishing technologies.

In conclusion, coarse Prunus spinosa biochar offers a robust, scalable, and economically viable solution for enhancing the resilience of nitrogen management in modern aquaculture. Future research should prioritize long-term, pilot-scale continuous flow trials under diverse RAS wastewater matrices to fully quantify the impacts of biofouling, hydraulic loading rates, and cumulative regeneration cycles on the material’s lifecycle performance. This study provides the necessary application-oriented foundation for such transitions, moving biochar-based treatment from fundamental batch observations toward resilient fixed-bed engineering reality.

## Data Availability

All data generated or analyzed during this study are included in this published article.

## References

[1] Pradeepkiran, J. A. Aquaculture role in global food security with nutritional value: a review. *Translational Anim. Sci.* **3**(2), 903–910 (2019). 10.1093/tas/txz012 PMC7200472 32704855

[2] Huang, Y. et al. Nitrogen cycling and resource recovery from aquaculture wastewater treatment systems: a review. *Environ. Chem. Lett.* **22**(5), 2467–2482 (2024).

[3] Kasper, S. et al. *Aquatic environment and life support systems*. Fundamentals of aquatic veterinary medicine. (2022).

[4] Kim, S. K. et al. Removal of ammonium-N from a recirculation aquacultural system using an immobilized nitrifier. *Aquacult. Eng.* **21**(3), 139–150 (2000).

[5] Ortiz, I. A. S. et al. Evaluation of acute toxicity of ammonia in Genetically Improved Farmed Tilapia. *Aquaculture Rep.* **27**, 101325 (2022).

[6] Preena, P. G., Rejish Kumar, V. J. & Singh, I. S. B. Nitrification and denitrification in recirculating aquaculture systems: the processes and players. *Reviews Aquaculture*. **13**(4), 2053–2075 (2021).

[7] John, E. M., Krishnapriya, K. & Sankar, T. Treatment of ammonia and nitrite in aquaculture wastewater by an assembled bacterial consortium. *Aquaculture* **526**, 735390 (2020).

[8] Sarosh, S. et al. Recirculating aquaculture system and nitrification: A review. *J. Indian Inst. Sci.* **104**(4), 869–892 (2024).

[9] Castellanos, R. M. et al. Effect of sludge age on aerobic granular sludge: Addressing nutrient removal performance and biomass stability. *Process Saf. Environ. Prot.* **149**, 212–222 (2021).

[10] Duarte, K. et al. Start-up of an aerobic granular sludge system from stored granules: Evaluating the impact of storage period on biomass activity and stability and the effect of temperature on nitrification and phosphorus removal rates. *J. Environ. Manage.* **296**, 113200 (2021). 34284343 10.1016/j.jenvman.2021.113200

[11] Li, W. et al. Contributions of nitrification and denitrification to N2O emissions from aged refuse bioreactor at different feeding loads of ammonia substrates. *Waste Manage.* **68**, 319–328 (2017). 10.1016/j.wasman.2017.06.037 28662844

[12] Torà, J. A. et al. Fast start-up and controlled operation during a long-term period of a high-rate partial nitrification activated sludge system. *Environ. Technol.* **33**(12), 1361–1366 (2012). 22856310 10.1080/09593330.2011.626802

[13] U.S. Environmental Protection Agency. Aquatic life ambient water quality criteria for ammonia – Freshwater 2013 (EPA-822-R-13-001). (Office of Water, 2013).

[14] Cao, L. et al. The application of post-denitrification fixed biofilm reactor for polishing secondary effluent: Nitrate removal, soluble microbial products and micropollutants biotransformation. *Bioresour. Technol.* **369**, 128511 (2023). 36538964 10.1016/j.biortech.2022.128511

[15] Wang, L. et al. Nitrogen removal for low concentration ammonium wastewater by adsorption, shortcut simultaneous nitrification and denitrification process in MBBR. *Water* **15**(7), 1334 (2023).

[16] Balasubramanian, S. et al. Review on process intensification for adsorptive wastewater treatment: Focus on bed geometries. *Adsorption* **31**(4), 64 (2025).

[17] Kato, S. & Kansha, Y. Comprehensive review of industrial wastewater treatment techniques. *Environ. Sci. Pollut. Res.* **31**(39), 51064–51097 (2024). 10.1007/s11356-024-34584-0 PMC11374848 39107648

[18] Trotochaud, L., Hawkins, B. T. & Stoner, B. R. Non-biological methods for phosphorus and nitrogen removal from wastewater: A gap analysis of reinvented-toilet technologies with respect to ISO 30500. *Gates Open. Res.* **3**, 559 (2020). 32494770 10.12688/gatesopenres.12931.1 PMC7232852

[19] Jiang, J. et al. Renewable, biomass-derived, honeycomblike aerogel as a robust oil absorbent with two-way reusability. *ACS Sustain. Chem. Eng.* **5**(11), 10307–10316 (2017).

[20] Sun, Q. M., He, J. H. & Lu, J. M. Recent advances and potential applications of flexible adsorption and separation materials: A review. *Energy Sci. Eng.* **11**(2), 952–973 (2023).

[21] da Conceição, F. T. et al. Biochar from sugarcane bagasse for reactive dye adsorption considering a circular economy approach. *J. Text. Eng. Fash Technol.* **8**, 126–132 (2022).

[22] Srivatsav, P. et al. Biochar as an eco-friendly and economical adsorbent for the removal of colorants (dyes) from aqueous environment: A review. *Water* **12**(12), 3561 (2020).

[23] Hafshejani, L. D. et al. Removal of nitrate from aqueous solution by modified sugarcane bagasse biochar. *Ecol. Eng.* **95**, 101–111 (2016).

[24] Haghighi Mood, S. et al. Iron-and nitrogen-modified biochar for nitrate adsorption from aqueous solution. *Sustainability* **16**(13), 5733 (2024).

[25] Kasera, N. et al. Synthesis, characterization, and use of magnesium-activated biochar for nitrate removal from aqueous solutions. *Int. J. Environ. Res.* **19**(5), 193 (2025).

[26] Thao, V. T. M. et al. Adsorption of ammonium, nitrite, and nitrate onto rice husk biochar for nitrogen removal. *Ho Chi Minh City Open. Univ. J. Science-Engineering Technol.* **11**(1), 29–43 (2021).

[27] Zhang, M. et al. Evaluating biochar and its modifications for the removal of ammonium, nitrate, and phosphate in water. *Water Res.* **186**, 116303 (2020). 32841930 10.1016/j.watres.2020.116303

[28] Fang, J. et al. Release and stability of water dispersible biochar colloids in aquatic environments: effects of pyrolysis temperature, particle size, and solution chemistry. *Environ. Pollut.* **260**, 114037 (2020). 32006888 10.1016/j.envpol.2020.114037

[29] Zhao, Q. et al. Preparation and application in water treatment of magnetic biochar. *Front. Bioeng. Biotechnol.* **9**, 769667 (2021). 34760880 10.3389/fbioe.2021.769667 PMC8572963

[30] Joshi, M., Bhatt, D. & Srivastava, A. Enhanced adsorption efficiency through biochar modification: A comprehensive review. *Ind. Eng. Chem. Res.* **62**(35), 13748–13761 (2023).

[31] Liu, Z. et al. Biochar particle size, shape, and porosity act together to influence soil water properties. *Plos one*. **12**(6), e0179079 (2017). 28598988 10.1371/journal.pone.0179079 PMC5466324

[32] Ahmad, M. et al. Biochar as a sorbent for contaminant management in soil and water: a review. *Chemosphere* **99**, 19–33 (2014). 24289982 10.1016/j.chemosphere.2013.10.071

[33] Ben-Natan, D., Fragman-Sapir, O. & Shemesh, B. Contributions to the Flora Palaestina Region, part 2. *Flora Mediterranea*. 35 (2025).

[34] Wang, X. et al. Recent advances in biochar application for water and wastewater treatment: A review. *PeerJ* **8**, e9164 (2020). 32477836 10.7717/peerj.9164 PMC7243815

[35] Foo, K. Y. & Hameed, B. H. Insights into the modeling of adsorption isotherm systems. *Chem. Eng. J.* **156**(1), 2–10 (2010).

[36] Ruthven, D. M. *Principles of Adsorption and Adsorption Processes* (Wiley, 1984).

[37] Timmons, M. B., Guerdat, T. & Vinci, B. J. *Recirculating Aquaculture* 4th edn (Ithaca Publishing Company LLC, 2018).

[38] Wheaton, F. W. *Aquacultural Engineering*. (1993).

[39] Udayakumar, R. et al. A system dynamics model for water quality management in recirculating aquaculture systems (Ras). *Nat. Eng. Sci.* **10**(2), 434–446 (2025).

[40] Noori, Z. et al. The effect of sediment particle size on the characteristics of phosphorus adsorption. *J. Environ. Stud.* **45**(3), 471–483 (2019).

[41] Tan, K. L. & Hameed, B. Insight into the adsorption kinetics models for the removal of contaminants from aqueous solutions. *J. Taiwan Inst. Chem. Eng.* **74**, 25–48 (2017).

[42] Tong, Y., McNamara, P. J. & Mayer, B. K. Adsorption of organic micropollutants onto biochar: A review of relevant kinetics, mechanisms and equilibrium.. *Environ. Sci. Water Res. Technol.* **5**(5), 821–838 (2019).

[43] Araújo, C. S. et al. Elucidation of mechanism involved in adsorption of Pb (II) onto lobeira fruit (Solanum lycocarpum) using Langmuir, Freundlich and Temkin isotherms. *Microchem. J.* **137**, 348–354 (2018).

[44] Saleh, T. A. Isotherm, kinetic, and thermodynamic studies on Hg (II) adsorption from aqueous solution by silica-multiwall carbon nanotubes. *Environ. Sci. Pollut. Res.* **22**(21), 16721–16731 (2015). 10.1007/s11356-015-4866-z 26087931

[45] Hamid, U. & Chen, C. C. Adsorption thermodynamics for process simulation. *Langmuir* **40**(45), 23583–23597 (2024). 39466233 10.1021/acs.langmuir.4c02579

[46] Le, H. et al. Size-dependent biochar breaking under compaction: Implications on clogging and pathogen removal in biofilters. *Environ. Pollut.* **266**, 115195 (2020). 32683234 10.1016/j.envpol.2020.115195

[47] Yatimi, Y., Alalou, A. & Al-Dahhan, M. Identification of flow regime in a co-current upflow packed bed reactor (U-PBR) using gamma-ray densitometry (GRD). *Chem. Eng. Sci.* **315**, 121871 (2025).

[48] Patel, H. Fixed-bed column adsorption study: A comprehensive review. *Appl. Water Sci.* **9**(3), 45 (2019).

[49] Rice, E. W. et al. *Standard methods for the examination of water and wastewater.* (2012).

[50] Blankenship, L. S., Jagiello, J. & Mokaya, R. Confirmation of pore formation mechanisms in biochars and activated carbons by dual isotherm analysis. *Mater. Adv.* **3**(9), 3961–3971 (2022).

[51] Du, L. et al. Evolution of biochar structure and its impact on volatile adsorption and reforming during char-recycled pyrolysis *Energy Mater. Adv.* **6**, 0167 (2025).

[52] Rady, O. et al. Synergistic adsorption and oxidation of arsenite by Fe–Mn binary oxide-modified bamboo biochar in the presence of air.. *Sci. Rep.*10.1038/s41598-025-32178-5 (2025). 41455736 10.1038/s41598-025-32178-5 PMC12749615

[53] Wang, J. & Wang, S. Preparation, modification and environmental application of biochar: A review. *J. Clean. Prod.* **227**, 1002–1022 (2019).

[54] Tan, X. et al. Application of biochar for the removal of pollutants from aqueous solutions. *Chemosphere* **125**, 70–85 (2015). 25618190 10.1016/j.chemosphere.2014.12.058

[55] Islam, I. U. et al. Kinetic studies and adsorptive removal of chromium Cr (VI) from contaminated water using green adsorbent prepared from agricultural waste, rice straw. *Eur. J. Chem.* **13**(1), 78–90 (2022).

[56] Li, X. & Shi, J. Simultaneous adsorption of tetracycline, ammonium and phosphate from wastewater by iron and nitrogen modified biochar: Kinetics, isotherm, thermodynamic and mechanism. *Chemosphere* **293**, 133574 (2022). 35016962 10.1016/j.chemosphere.2022.133574

[57] Ofomaja, A. E., Naidoo, E. B. & Pholosi, A. Intraparticle diffusion of Cr (VI) through biomass and magnetite coated biomass: A comparative kinetic and diffusion study. *S. Afr. J. Chem. Eng.* **32**(1), 39–55 (2020).

[58] Abiodun, O. A. O. et al. Remediation of heavy metals using biomass-based adsorbents: adsorption kinetics and isotherm models. *Clean. Technol.* **5**(3), 934–960 (2023).

[59] Wang, S. et al. A study on and adsorption mechanism of ammonium nitrogen by modified corn straw biochar.. *R. Soc. Open Sci.*10.1098/rsos.221535 (2023). 36778959 10.1098/rsos.221535 PMC9905994

[60] Caplan, J. S. et al. Coarse biochar improves the hydraulic performance of compacted roadside soil media. *J. Sustain. Water Built Environ.* **11**(4), 04025010 (2025).

[61] Sun, M. et al. Porous biochar adsorbent prepared by cold isostatic pressure pretreatment and adsorption performance of Cr (VI) at C and N dual active sites.. *J. Environ. Chem. Eng.*10.1016/j.jece.2025.118286 (2025).

[62] Cai, L. F. et al. Structural control of a novel hierarchical porous carbon material and its adsorption properties. *Sci. Rep.* **12**(1), 3118 (2022). 35210445 10.1038/s41598-022-06781-9 PMC8873302

[63] Lee, W. & Choi, Y. Ammonia recovery and valorization from wastewater via adsorption, regeneration, and refining technologies. *Environ. Eng. Res.* **31**(2), 10–35 (2026).

[64] Wang, B., Gao, B. & Fang, J. Recent advances in engineered biochar productions and applications. *Crit. Rev. Environ. Sci. Technol.* **47**(22), 2158–2207 (2017).

[65] Van Rijn, J. Waste treatment in recirculating aquaculture systems. *Aquacult. Eng.* **53**, 49–56 (2013).

[66] Trazzi, P. A. et al. Adsorption of ammonium, nitrate, and phosphate on hydrochars and biochars. *Appl. Sci.* **14**(6), 2280 (2024).

[67] Zhu, Z. et al. Stability of functionally modified biochar: The role of surface charges and surface homogeneity. *Sustainability* **15**(10), 7745 (2023).

[68] Ernst, A. et al. Virtual sensing of nitrite: A novel control for safe denitrification in recirculating aquaculture systems (RASs). *Fishes* **9**(10), 398 (2024).

[69] He, Z. et al. Effects of biochar particle size on sorption and desorption behavior of NH4+-N. *Ind. Crops Prod.* **189**, 115837 (2022).

[70] Fan, Q. et al. Effects of chemical oxidation on surface oxygen-containing functional groups and adsorption behavior of biochar. *Chemosphere* **207**, 33–40 (2018). 29772422 10.1016/j.chemosphere.2018.05.044

[71] Mel’gunov, M. S. Application of the simple Bayesian classifier for the N2 (77 K) adsorption/desorption hysteresis loop recognition. *Adsorption* **29**(5), 199–208 (2023).

[72] Eding, E. et al. Design and operation of nitrifying trickling filters in recirculating aquaculture: a review. *Aquacult. Eng.* **34**(3), 234–260 (2006).

[73] Taslakyan, L. et al. Biochar-integrated reactive filtration of wastewater for P removal and recovery, micropollutant catalytic oxidation, and negative CO2e: Life cycle assessment and techno-economic analysis. *Water Environ. Res.* **95**(12), e10962 (2023). 38153197 10.1002/wer.10962

[74] Zhu, Y. et al. Evaluating biochar for adsorption of ammonium nitrogen in wastewater: insights into modifications and mechanisms. *Environ. Res.* **277**, 121615 (2025). 40239738 10.1016/j.envres.2025.121615

[75] Edet, U. A. & Ifelebuegu, A. O. Kinetics, isotherms, and thermodynamic modeling of the adsorption of phosphates from model wastewater using recycled brick waste. *Processes* **8**(6), 665 (2020).

[76] Guo, X. & Wang, J. A general kinetic model for adsorption: Theoretical analysis and modeling. *J. Mol. Liq.* **288**, 111100 (2019).

[77] Keramatzadeh, M., Ehteshami, M. & Takdastan, A. Efficient removal of 2, 4-D using NaOH-activated date seed biochar with adsorption behavior, kinetic and isotherm modeling, and process optimization.. *Sci. Rep.*10.1038/s41598-025-30144-9 (2025). 41326613 10.1038/s41598-025-30144-9 PMC12775533

[78] Gutiérrez, M., Verlicchi, P. & Mutavdžić Pavlović, D. Study of the influence of the wastewater matrix in the adsorption of three pharmaceuticals by powdered activated carbon.. *Molecules* **28**(5), 2098 (2023). 36903344 10.3390/molecules28052098 PMC10004314

[79] Kizito, S. et al. Evaluation of ammonium adsorption in biochar-fixed beds for treatment of anaerobically digested swine slurry: experimental optimization and modeling. *Sci. Total Environ.* **563**, 1095–1104 (2016). 27241205 10.1016/j.scitotenv.2016.05.149

[80] Wu, X. et al. Adsorption characteristics and mechanism of ammonia nitrogen and phosphate from biogas slurry by Ca2+-modified soybean straw biochar. *PLoS One* **18**(8), e0290714 (2023). 37624822 10.1371/journal.pone.0290714 PMC10456179

[81] Zhang, Y. et al. Production of biochar from lignocellulosic biomass with acidic deep eutectic solvent and its application as efficient adsorbent for Cr (VI). *J. Clean. Prod.* **324**, 129270 (2021).

[82] Wang, Y. et al. Research status, trends, and mechanisms of biochar adsorption for wastewater treatment: a scientometric review. *Environ. Sci. Europe*. **36**(1), 25 (2024).

[83] Xu, H. et al. Adsorption behavior and performance of ammonium onto sorghum straw biochar from water. *Sci. Rep.* **12**(1), 5358 (2022). 35354834 10.1038/s41598-022-08591-5 PMC8967861

[84] Gence, C. Ç. & Erdem, H. Determination of NH4+ and NO3-adsorption and desorption capacities of biochars produced at different temperatures. *Turk. J. Agric. - Food Sci. Technol.* **13**(4), 985–990 (2025).

[85] Aghoghovwia, M. P., Hardie, A. G. & Rozanov, A. B. Characterisation, adsorption and desorption of ammonium and nitrate of biochar derived from different feedstocks. *Environ. Technol.* **43**(5), 774–787 (2022). 32741271 10.1080/09593330.2020.1804466

[86] Girish, C. R. Determination of thermodynamic parameters in adsorption studies: a review. *Chem. Pap.* **79**(9), 5687–5706 (2025).

[87] Saha, N. et al. Comparative experimental and mathematical analysis on removal of dye using raw rice husk, rice husk charcoal and activated rice husk charcoal: batch, fixed-bed column, and mathematical modeling. *Biomass Convers. Biorefinery*. **13**(12), 11023–11040 (2023).

[88] de Oliveira, A. S. S. et al. Recycling nitrogen with water lettuce biochar: ammonium adsorption from industrial wastewater and application as soil conditioner. *Environ. Earth Sci.* **84**(21), 1–16 (2025).

[89] Wang, M. et al. Biochar production using biogas residue and their adsorption of ammonium nitrogen and chemical oxygen demand in wastewater. *Biomass Convers. Biorefinery*. **13**(5), 3881–3892 (2023).

[90] Saidu, M. M. et al. Efficient temperature control in recirculating aquaculture tanks. *Appl. Eng. Agric.* **28**(1), 161–167 (2012).

[91] Abbas, Z. et al. A critical review of mechanisms involved in the adsorption of organic and inorganic contaminants through biochar. *Arab. J. Geosci.* **11**(16), 448 (2018).

[92] Wang, B. et al. Adsorption and desorption of ammonium by maple wood biochar as a function of oxidation and pH. *Chemosphere* **138**, 120–126 (2015). 26057391 10.1016/j.chemosphere.2015.05.062

[93] Atif, M. et al. Physisorption and chemisorption trends in surface modification of carbon black. *Surf. Interfaces*. **31**, 102080 (2022).

[94] Mbugua, J. K. et al. The role of biochar in sustainable wastewater management in Kenya: current practices, challenges, and future prospects. *J. Environ. Ecol.* **1**(1), 10–16 (2025).

[95] Wang, Z. & Sedighi, M. Disintegration of biochar adsorbent under the hydraulic conditions of fixed bed water treatment. *Chemosphere* **336**, 139294 (2023). 37353173 10.1016/j.chemosphere.2023.139294

[96] Knox, J. C. et al. Limitations of breakthrough curve analysis in fixed-bed adsorption. *Ind. Eng. Chem. Res.* **55**(16), 4734–4748 (2016). 31359909 10.1021/acs.iecr.6b00516 PMC6662230

[97] Syeda, H. I., Muthukumaran, S. & Baskaran, K. Dynamic adsorption of heavy metals on functionalized and regeneratable biopolymeric aerogels: Fixed-bed column reactor modelling and dual functionality elution technique. *Sep. Purif. Technol.* **363**, 131861 (2025).

[98] Poursaeidesfahani, A. et al. Prediction of adsorption isotherms from breakthrough curves. *Microporous Mesoporous Mater.* **277**, 237–244 (2019).

[99] Yapsakli, K., Aktan, C. K. & Mertoglu, B. Anammox-zeolite system acting as buffer to achieve stable effluent nitrogen values. *Biodegradation* **28**(1), 69–79 (2017). 27807679 10.1007/s10532-016-9778-1

[100] Zhang, Z. & Shu, Y. Simulation of fixed-bed adsorption process considering particle size distribution. *Chin. J. Chem. Eng.* **74**, 175–189 (2024).

[101] Alrbai, M. et al. Techno-economic feasibility study of ammonia recovery from sewage sludge digestate in wastewater treatment plants. *Clean. Environ. Syst.* **15**, 100235 (2024).

[102] Behjat, M. et al. Life cycle assessment of recirculating aquaculture systems with innovative biochar filter for enhanced nutrient recirculation. *Resour. Environ. Sustain.* **21**, 100233 (2025).

[103] Yang, H. I. et al. Adsorption of ammonium in aqueous solutions by pine sawdust and wheat straw biochars. *Environ. Sci. Pollut. Res.* **25**(26), 25638–25647 (2018). 10.1007/s11356-017-8551-2 28229381

[104] Vu, T. M. et al. Removing ammonium from water using modified corncob-biochar. *Sci. Total Environ.* **579**, 612–619 (2017). 27890415 10.1016/j.scitotenv.2016.11.050

[105] Bellahsen, N. et al. Pomegranate peel as a new low-cost adsorbent for ammonium removal. *Int. J. Environ. Sci. Technol.* **18**(3), 711–722 (2021).

[106] Nguyen, L. H. et al. Ammonium removal from aqueous solutions by fixed-bed column using corncob-based modified biochar. *Environ. Technol.* **40**(6), 683–692 (2019). 29161983 10.1080/09593330.2017.1404134

[107] Kavindi, G. A. G., Tang, L. & Sasaki, Y. Assessing GHG emission reduction in biomass-derived biochar production via slow pyrolysis: A cradle-to-gate LCA approach. *Resour. Conserv. Recycl.* **212**, 107900 (2025).

[108] Dawood, I. et al. Sustainable water purification through the adsorptive removal of tetracycline via modified clay and regeneration via controlled thermolysis: A reusable and environmentally friendly approach for contaminant mitigation. *Appl. Water Sci.* **15**(8), 215 (2025).

[109] Gkika, D. A. & Kyzas, G. Z. Reusability of spent adsorbents for a circular materials economy in the sustainable chemical industry.. *RSC Sustain.*10.1039/D5SU00802F (2026).

[110] Yao, A. K. Z. et al. Ammonium sorption and regeneration using Mg-modified zeolites: A study on the interferences of competing ions from aquaculture effluent. *J. Water Process. Eng.* **48**, 102909 (2022).

[111] Judd, S. *The MBR Book: Principles and Applications of Membrane Bioreactors for Water and Wastewater Treatment* (Elsevier, 2010).

[112] Van der Bruggen, B. et al. A review of pressure-driven membrane processes in wastewater treatment and drinking water production. *Environ. Prog.* **22**(1), 46–56 (2003).

[113] Fang, L., Xing, Y. & Han, Y. *Technical and Economic Analysis of Biochar Technologies*. Waste-derived biochar for sustainable rural revitalization, 407–424 (2025).

